# An Updated Checklist of Cadaver-Associated Culturable Fungi, Along with the Isolation of Microfungi from a 70-Year-Old Male Cadaver

**DOI:** 10.3390/jof12100780

**Published:** 2026-10-18

**Authors:** Marco Iannaccone, Laura Canonica, Silvia Giusto, Grazia Cecchi, Simone Di Piazza, Rosario Barranco, Francesco Ventura, Mirca Zotti

**Affiliations:** 1 Laboratory of Mycology, Department of Earth, Environment and Life Science, University of Genoa, Corso Europa 26, 16132 Genoa, Italy; marco.iannaccone@edu.unige.it (M.I.); laura.canonica95@gmail.com (L.C.); silvia.giusto@edu.unige.it (S.G.); grazia.cecchi@unige.it (G.C.); mirca.zotti@unige.it (M.Z.); 2 Department of Legal and Forensic Medicine, University of Genoa, Via De Toni 12, 16132 Genoa, Italy; rosario.barranco@unige.it (R.B.); francesco.ventura@unige.it (F.V.)

**Keywords:** forensic mycology, cadaver-associated fungi, culturable fungi, DNA barcoding, postmortem interval, fungal succession, forensic taphonomy

## Abstract

Forensic mycology investigates the role of fungi in human decomposition and may provide useful information on the postmortem interval (PMI), environmental conditions, and possible body movement. However, cadaver-associated fungal communities remain insufficiently investigated, particularly in terms of culturable taxa and their preservation in reference collections. To address this gap, we present an updated checklist of cadaver-associated culturable fungi spanning 1923–2026, compiled from 27 literature sources across 12 countries and comprising 883 records (879 from the literature and 4 from this study), including previously overlooked early 20th-century Italian records recovered from historical archives. The checklist includes four new isolates obtained from a 70-year-old male cadaver found in a basement in the putrefaction stage. Visible fungal growth was sampled from the left hand, left cheek, and right calf, cultured on Sabouraud and Rose Bengal media, and identified through morphological analysis and DNA barcoding: *Penicillium brevicompactum*, *Penicillium chrysogenum*, *Mucor saturninus*, and *Aspergillus versicolor*. Notably, *A. versicolor* was recovered from a cadaver in the putrefaction stage, expanding current knowledge of its forensic relevance. All isolates were deposited in the ColD UNIGE JRU MIRRI-IT collection, contributing to the development of forensic mycological databases.

## 1. Introduction

Forensic taphonomy aims to understand the processes involved in decomposition, the factors influencing decomposition stages, the cause of death, and the estimation of the postmortem interval (PMI) [1]. Decomposition is commonly divided into bloating, putrefaction, and skeletonization, although further subdivisions have been proposed [2,3]. These stages are investigated by several biological disciplines, including forensic mycology [4,5,6,7]. The mycelium of saprotrophic fungi secretes enzymes that degrade complex organic matter, including cadaveric tissues [8,9]. Saprotrophic fungi act as decomposers by feeding on dead organic matter and recycling carbon and other nutrients [10]. Moreover, inoculum availability, together with abiotic and biotic environmental conditions, influences cadaver-associated fungal communities [2,7,11]. Over time, the necro-mycobiome has been studied using macro- and micromorphological characteristics and, more recently, DNA barcoding [12]. As reported by several authors, cadaveric fungi can provide relevant forensic information, including environmental conditions, PMI, and possible body movement [6,11]. Nevertheless, fungi are often neglected in forensic contexts, including with respect to the anatomical location of sampling on the body [6,11].

Studies on cadaver-associated fungi are increasing, as shown by recent publications, despite the difficulty of obtaining access to cadavers [2,6,7,13,14,15]. However, the number of culturable fungal species identified on cadavers remains limited: according to Piepenbring et al. [7], only 104 species have been listed. Moreover, considering current estimates of total fungal diversity [16], forensic mycology is still far from having a comprehensive dataset. The present work aimed to investigate the occurrence of fungi on a cadaver at the putrefaction stage and to contribute to forensic mycology through DNA barcoding confirmed records. An additional aim was to enrich the DISTAV culture collection (ColD-UNIGE JRU MIRRI-IT), a member and project partner of SUS-MIRRI.IT, by opening a section dedicated to a forensic mycological database.

## 2. Materials and Methods

### 2.1. Sampling of Fungi

**Figure 1:**
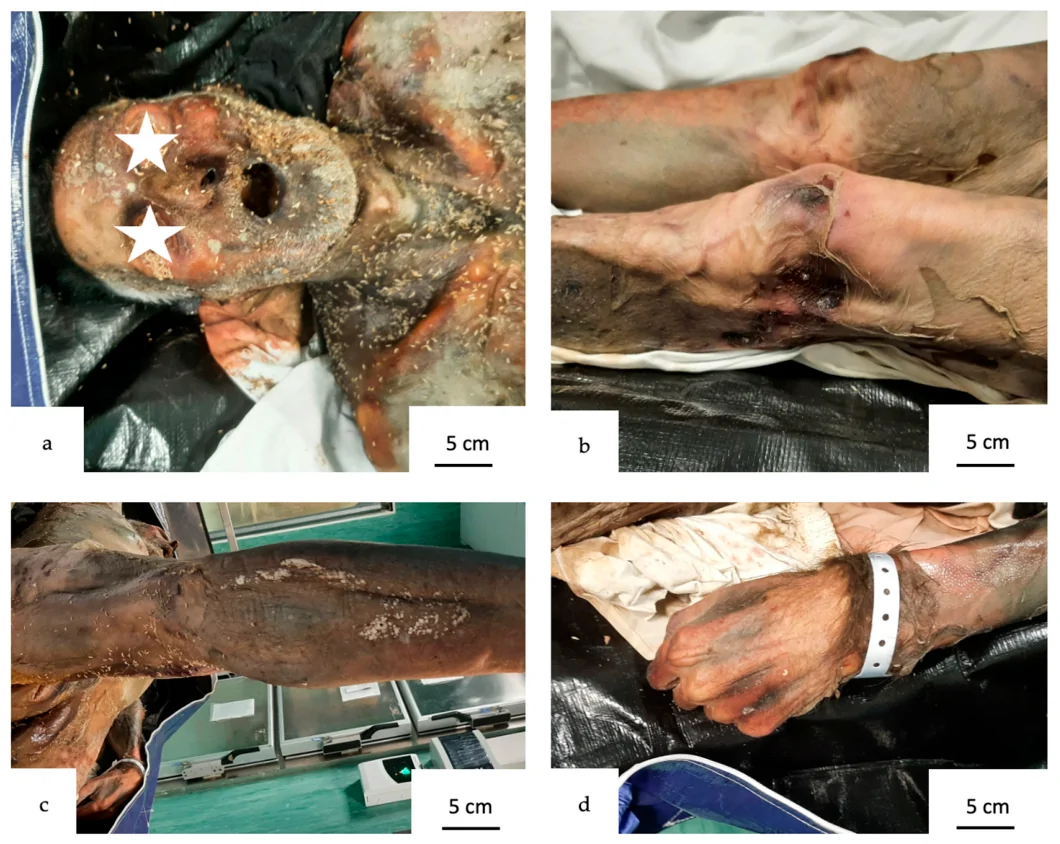
A cadaver at the putrefaction stage. Visible fungal colonies on the cheek (**a**), right calf (**b**,**c**), and left hand (**d**). Scale bars = 5 cm.

Samples were collected from a 70-year-old male cadaver found in a basement at the putrefaction stage. Three anatomical sites were sampled: the left hand, left cheek, and right calf (Table 1). Sampling areas were selected after visible fungal colonies were observed on the skin, as shown in Figure 1. No signs of trauma were detected and there was no suspicion of substance abuse or poisoning; owing to the advanced putrefactive changes, the cause of death was attributed to natural causes, most probably senile cardiac death, since colliquation and disintegration of the internal organs prevented a detailed anatomopathological examination. Based on the findings at examination (dipteran larvae at instars I–II with a few specimens at instar III, a diffuse putrefactive venous network over the whole body, a globular abdomen due to gas accumulation, tanned, leather-like skin on the head, thorax, back and hands, colliquation of the eyeballs, and widespread epidermolysis of the trunk), the postmortem interval (PMI) was estimated at approximately two weeks to three months. The body was transferred to the morgue on 15 January 2023. No on-site measurements of temperature or relative humidity were available for the basement; as a general indication of the ambient conditions over the estimated PMI window (mid-October 2022 to 15 January 2023), outdoor data recorded at the Genoa Sestri Ponente weather station show a progressive autumn-to-winter cooling: mean daily temperatures averaged 20.6 °C in 15–31 October, 14.1 °C in November, 10.3 °C in December and 12.9 °C in 1–15 January, with mean relative humidity of 80%, 66%, 74% and 73%, respectively (whole window: mean temperature 13.8 °C, daily minima and maxima between 2 and 25 °C, mean relative humidity 73%) [17]. To protect the researchers’ health and safety, biological masks, disposable caps, gowns, gloves, and protective footwear were worn throughout the procedure [18]. The postmortem examination was carried out in a BSL-3 autopsy room, in accordance with the Italian Police Mortuary Regulation (Presidential Decree no. 285 of 10 September 1990), using full personal protective equipment (coverall, gloves, shoe covers, FFP2 mask and face shield).

### 2.2. Fungal Isolation and Preservation

**Figure 2:**
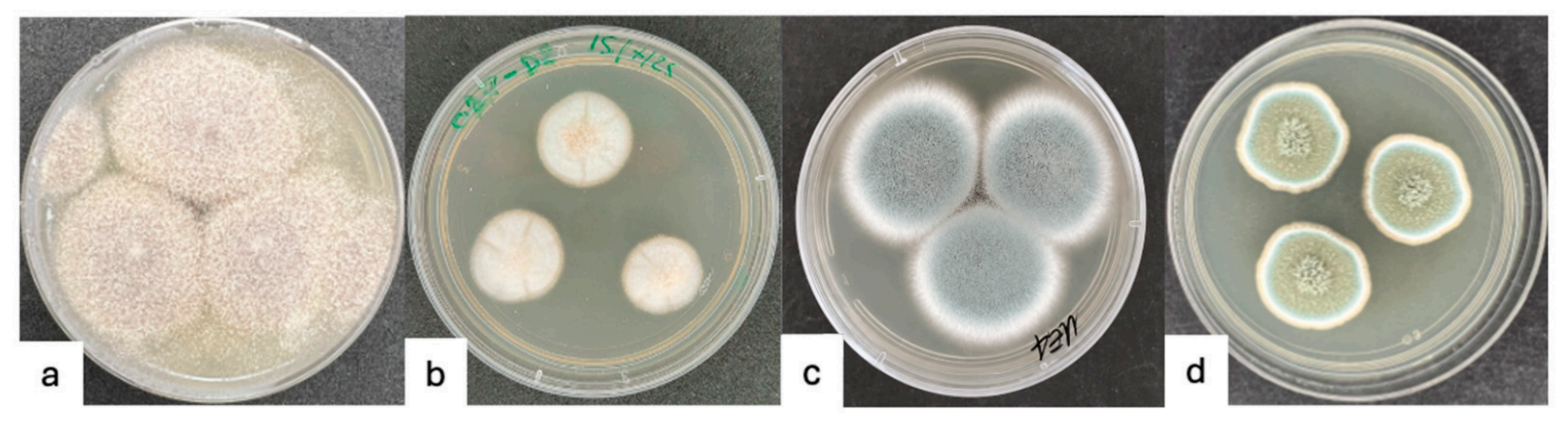
Petri dish cultures (6 cm ∅) of the four fungal isolates obtained from the cadaver: (**a**) *Mucor saturninus*; (**b**) *Aspergillus versicolor*; (**c**) *Penicillium chrysogenum*; (**d**) *Penicillium brevicompactum*.

Samples were collected from the above-mentioned areas using sterile scalpels and plated on Sabouraud (SAB) and Rose Bengal (RB) media. Once fungal colonies had developed sufficiently, serial subcultures were performed until each isolate was considered axenic. Colony growth pattern, color, and reverse color were recorded for each isolate (Figure 2). Micromorphological features were observed under a stereomicroscope and a light microscope using fragments of colonies collected aseptically from the conidiation area. Axenic isolates were stored in cryovials containing malt extract broth and glycerol (30%) at −20 °C and deposited in the ColD-UNIGE MIRRI-IT culture collection. All laboratory procedures involving fungal cultures were carried out in a Biosafety Level 2 (BSL-2) facility at the Laboratory of Mycology, DISTAV, University of Genoa, in accordance with the institutional biosafety and public health regulations in force at the University of Genoa.

### 2.3. Molecular Identification of the Isolates

Preliminary sorting of the isolated strains was carried out based on phenotypic characters. DNA barcoding was performed by amplifying the internal transcribed spacer (ITS) region using the fungal specific primer pair V9G (5′-TTACGTCCCTGCCCTTTGTA-3′) and LS266 (5′-GCATTCCCAAACAACTCGACTC-3′) [19,20,21]. Since genomic DNA was extracted from axenic fungal cultures obtained after serial subculturing on solid media (Section 2.2), rather than directly from cadaveric tissue, contamination with host (human) DNA was not a concern, and no blocking primers were required. Genomic DNA was extracted from 3-day-old cultures grown on Malt Extract Agar (MEA) using the Qiagen DNeasy Ultraclean Microbial DNA Isolation Kit (Qiagen, Hilden, Germany), according to the manufacturer’s instructions. The polymerase chain reaction (PCR) reaction contained 12 μL of mix (8.23 μL Milli-Q water (Merck Millipore, Burlington, MA, USA), 0.38 μL 50 mM MgCl_2_, 1.25 μL NH 10× PCR buffer taq, 0.98 μL 1 mM dNTPs (Dominique Dutscher SAS, Bernolsheim, France), 0.63 μL DMSO, 0.25 μL 10 μM of each primer, 0.05 μL BioTaq (Bioline, London, UK) 5 U/μL) and 1 μL of DNA template; these volumes refer to a single reaction, with a Master Mix proportionally scaled for the total number of samples processed in each run and then aliquoted into individual tubes to allow for accurate pipetting of the combined volumes. The PCR program was as follows: 5 min at 95 °C; 30–35 cycles of 35 s at 95 °C, 50 s at 55 °C, and 2 min at 72 °C; 5 min at 72 °C; and a final hold at 10 °C. Amplicons were bidirectionally sequenced, assembled, and compared using BLAST (Basic Local Alignment Search Tool version 2.17.0 (released in July 2025)) against sequences in the National Center for Biotechnology Information (NCBI) GenBank database [22]. A similarity threshold higher than 97% was considered preliminary support for species-level identification, in line with previously proposed criteria for ITS-based species level discrimination in fungi [23], together with morphological characters and, when available, additional loci. For isolates whose ITS sequence alone did not allow for unambiguous species-level resolution (Table 2), additional loci were amplified in separate PCR reactions using the same reaction mix and thermal cycling conditions described above, but with locus-specific primer pairs: the large subunit (LSU) of the nuclear ribosomal RNA gene was amplified with primers LR0R (5′-ACCCGCTGAACTTAAGC-3′) and LR5 (5′-TCCTGAGGGAAACTTCG-3′) [24], the partial β-tubulin (BT) gene with primers Bt2a (5′-GGTAACCAAATCGGTGCTGCTTTC-3′) and Bt2b (5′-ACCCTCAGTGTAGTGACCCTTGGC-3′) [25], and the partial calmodulin (CMD) gene with primers CMD5 (5′-CCGAGTACAAGGARGCCTTC-3′) and CMD6 (5′-CCGATRGAGGTCATRACGTGG-3′) [26]. Resulting sequences were analyzed as described above for ITS. For isolate 026-E4 (*Mucor saturninus*), no ITS sequence is available; its identification relies on the LSU sequence (Table 2) together with morphological characters. Accession numbers of all sequences generated in this study are reported in Table 1.

### 2.4. Literature Review and Checklist Compilation

**Figure 3:**
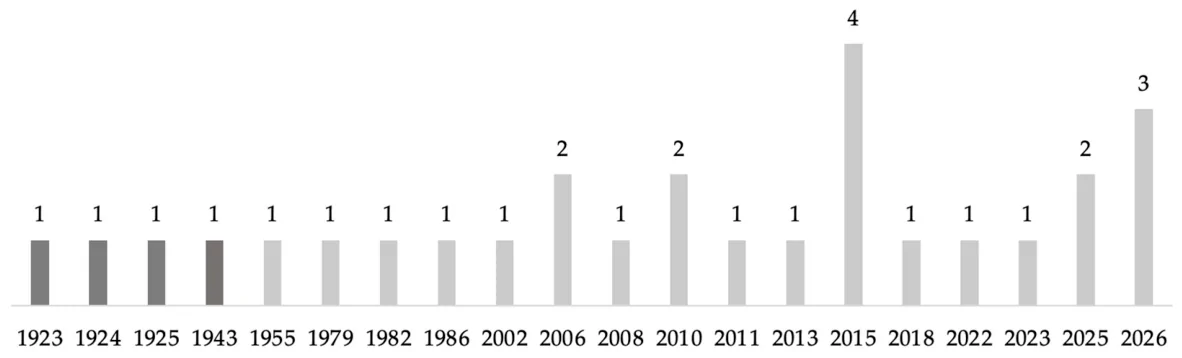
The number of publications related to forensic mycology from 1923 to 2026. Bar height indicates the number of papers published in that year, not the number of fungal records. Dark gray marks the three earliest sources, Bianchini (1923) [27], Bianchini and Manfrini (1924) [28], Bianchini (1925) [29], and Ciferri (1943) [30] recovered from historical Italian archives and newly incorporated into the checklist, together contributing 50 of the 883 records analyzed in Figure 4, Figure 5, Figure 6 and Figure 7.

**Figure 4:**
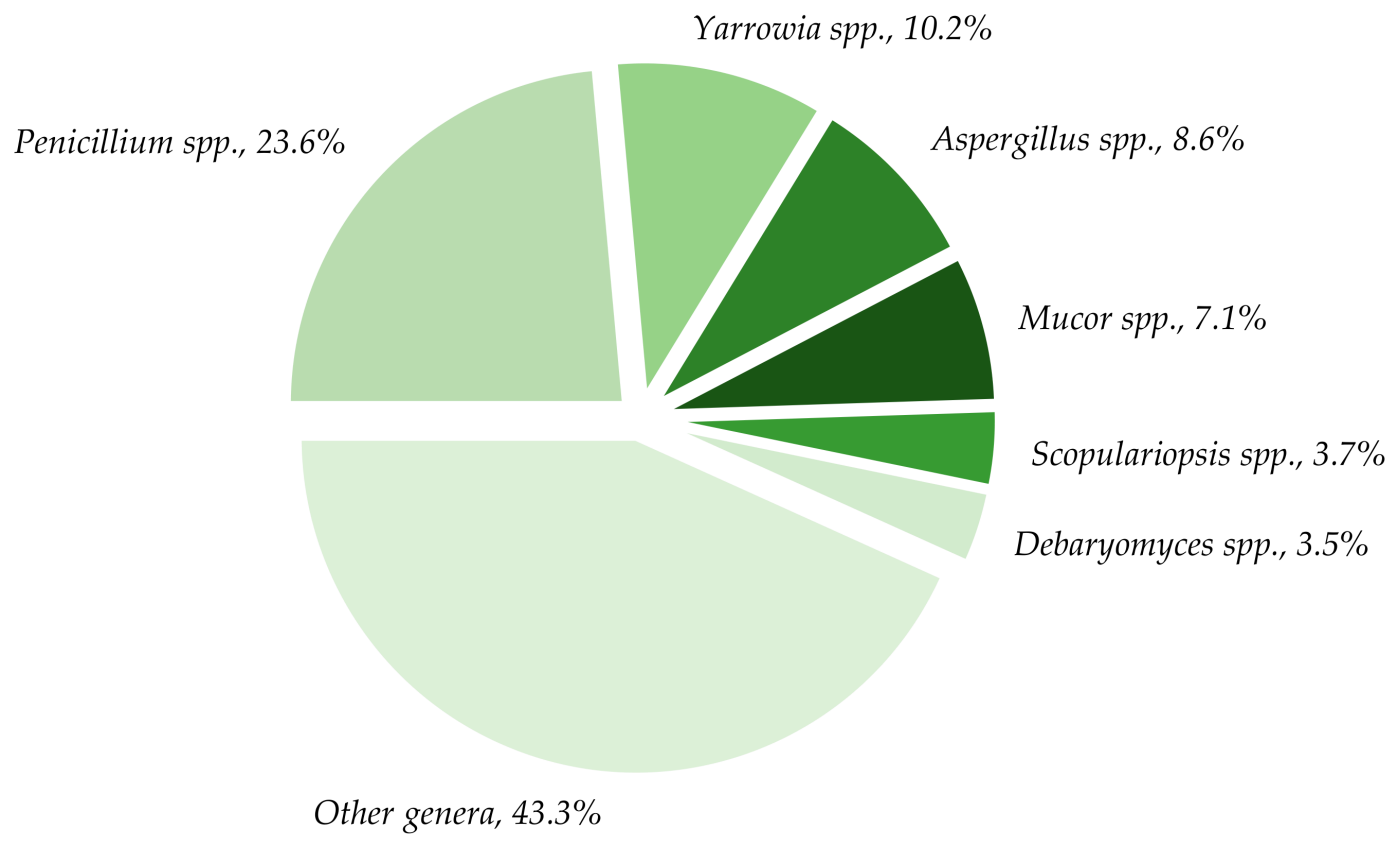
The number of culturable fungal isolates recorded from cadavers between 1923 and 2026, grouped by predominant genera (total = 883 records; 125 genera identified, plus 1 record for which the genus could not be assigned). The six genera each accounting for at least 3% of the records are shown individually: *Penicillium* spp. (208 records, 23.6%), *Yarrowia* spp. (90, 10.2%), *Aspergillus* spp. (76, 8.6%), *Mucor* spp. (63, 7.1%), *Scopulariopsis* spp. (33, 3.7%), and *Debaryomyces* spp. (31, 3.5%). The remaining 119 genera, each individually accounting for less than 3% of total records, are pooled into “Other genera” (382 records, 43.3%, including the record of unassigned genus) to improve chart readability. This category spans moderately represented genera such as *Pseudogymnoascus* (25 records), *Fusarium* (22), *Geotrichum* (16), *Cladosporium* (14), and *Talaromyces* (14), and a long tail of rarely reported taxa: 57 of the 119 pooled genera are represented by a single record each.

**Figure 5:**
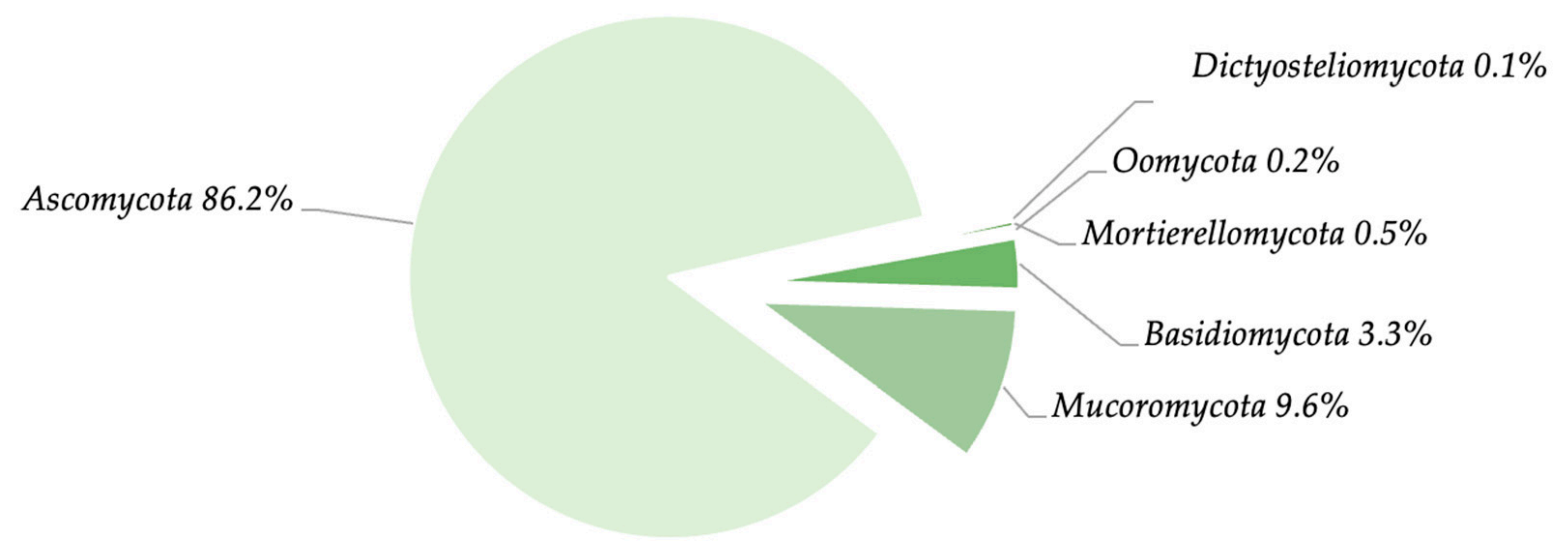
Relative abundance of fungal phyla among the 883 cadaver-associated isolates in the checklist: *Ascomycota* (86.2%), *Mucoromycota* (9.6%), *Basidiomycota* (3.3%), *Mortierellomycota* (0.5%), *Oomycota* (0.2%), and *Dictyosteliomycota* (0.1%). One additional record (0.1%) could not be assigned to a phylum.

**Figure 6:**
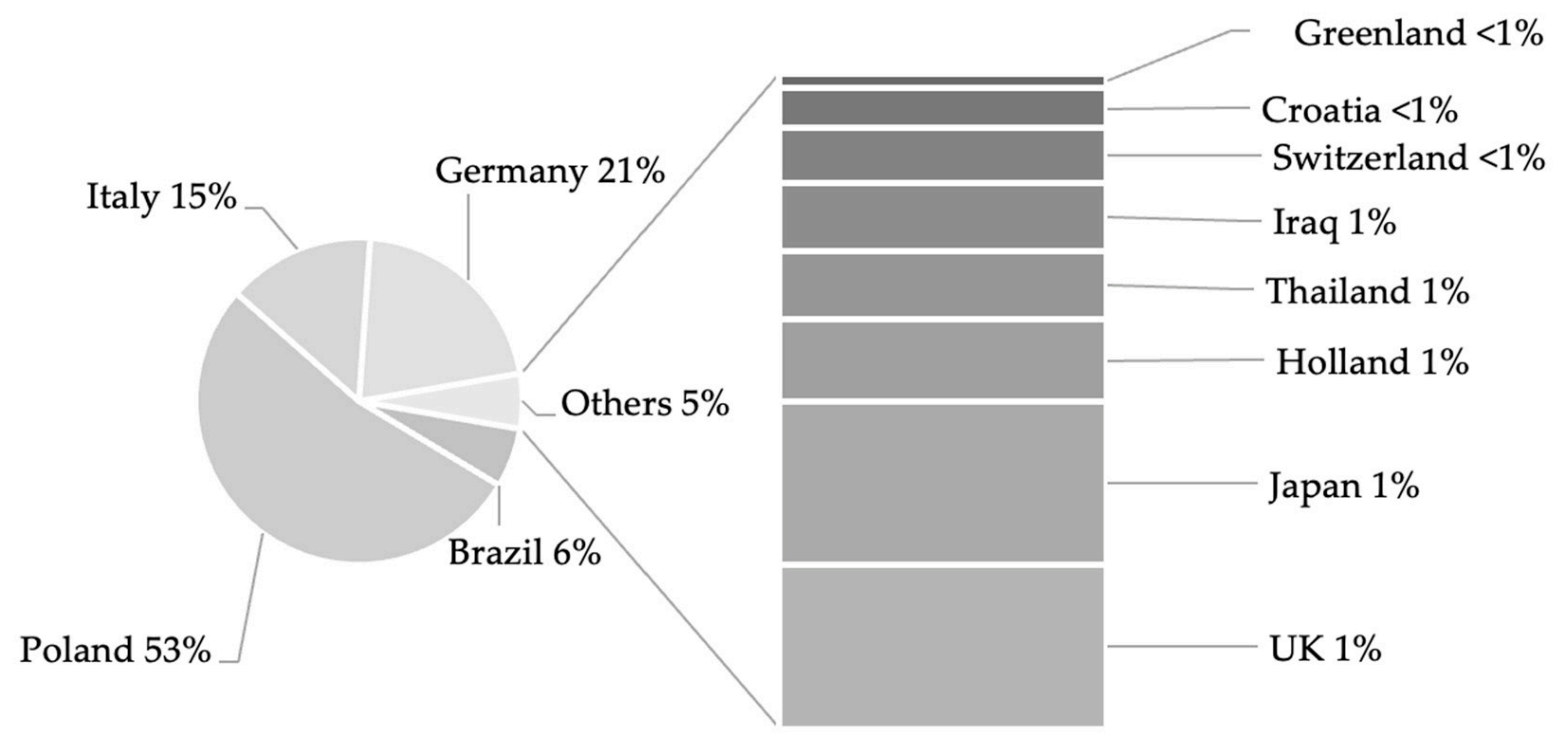
The percentage of fungal isolates reported from each of the 12 countries included in the checklist: Poland (52.9%), Germany (21.2%), Italy (14.6%), Brazil (5.9%), Japan (1.4%), the United Kingdom (1.4%), Holland (0.7%), Iraq (0.6%), Thailand (0.6%), Switzerland (0.5%), Croatia (0.3%), and Greenland (0.1%) (total = 883).

**Figure 7:**
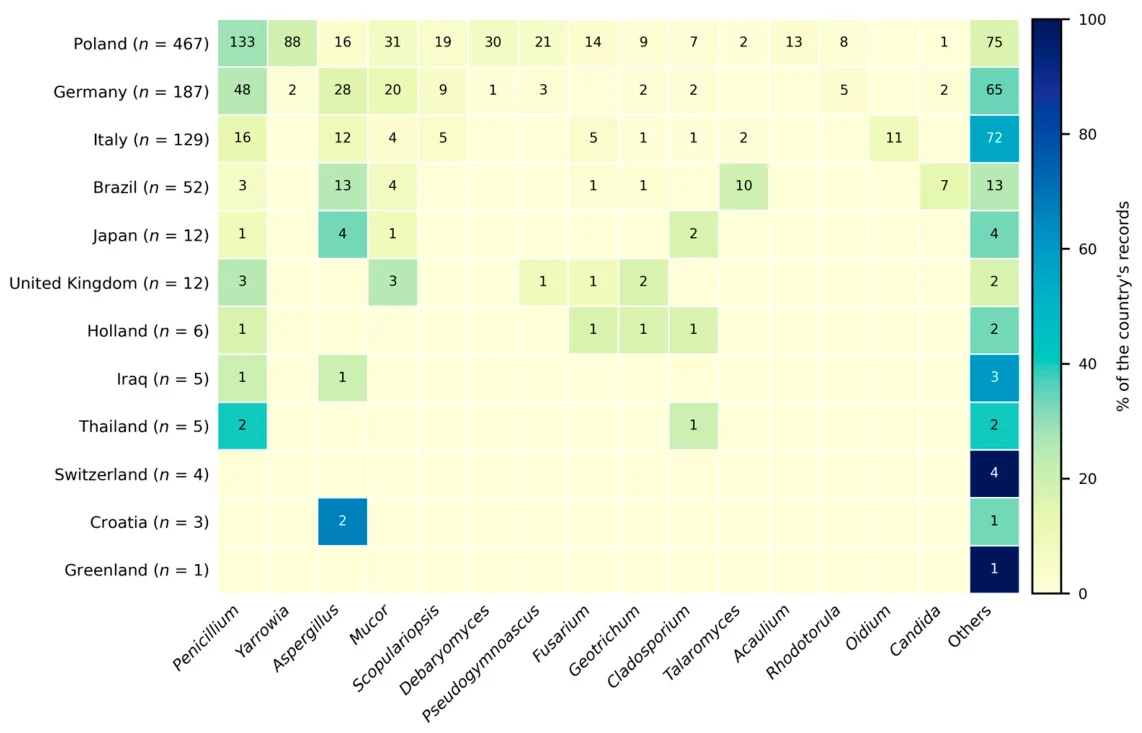
Genus composition of cadaver-associated fungal isolates by country. The heatmap shows, for each of the 12 countries included in the checklist (rows, ordered by number of records), the percentage of that country’s records (color scale) belonging to each of the 15 genera most frequently reported overall (columns); numbers within cells are record counts, and all remaining genera are pooled as “Others”. Percentages are calculated relative to each country’s own total number of records (*n*). Genus richness ranges from a single genus in Greenland (1 record) to 55 genera in Italy (129 records) and 46 in Poland (467 records).

An updated checklist of cadaver-associated culturable fungi was compiled by searching the peer-reviewed and historical literature published between 1923 and 2026 (Figure 3), building on and expanding the dataset previously reported by Piepenbring et al. [7]. Records were retrieved from scientific databases (Google Scholar, PubMed, Scopus) and from historical Italian mycological archives [27,28,29,30], using combinations of the terms “cadaver”, “corpse”, “forensic mycology”, “necro-mycobiome”, and “culturable fungi”. Studies were included when they reported culturable fungal taxa isolated from human cadavers or human remains, with an identifiable sampling country, decomposition stage, and literature source; studies based exclusively on culture-independent methods (e.g., metabarcoding, metagenomics) were excluded from the checklist, as they are not directly comparable to culture-dependent records. For each record, the taxon name reported in the original source was checked against current nomenclature using MycoBank (https://www.mycobank.org); names not currently accepted were updated to their valid name, and the original and validated names were both retained. The compiled checklist, comprising 883 records (879 from 27 literature sources across 12 countries, plus the 4 isolates obtained in the present study), is provided in Table 3. For each record, the genus, phylum, and country of origin were extracted from the compiled checklist (Table 3) and used to calculate relative frequencies (percentages of the total 883 records) at the genus, phylum, and country level; these frequencies are summarized in Figure 4, Figure 5, Figure 6 and Figure 7. Figure 3 shows the temporal distribution of the literature sources by year of publication, irrespective of the number of records each source contributed. In Figure 4, genera each accounting for less than 3% of the total records were pooled into a single ‘other genera’ category to improve the readability of the chart; countries and phyla are instead shown individually in Figure 5, Figure 6 and Figure 7, including those contributing less than 1% of records. In Figure 7, genus percentages were calculated relative to each country’s own total number of records rather than to the overall total of 883, so that color intensity reflects the relative abundance of each genus within that country; one record for which the phylum could not be resolved is excluded from Figure 5 only.

## 3. Results

A total of four isolates were obtained from the cadaver. The isolates from the left hand and left cheek were identified as *Penicillium brevicompactum* (Figure 2d) and *Penicillium chrysogenum* (Figure 2c), respectively. *Mucor saturninus* (Figure 2a) was also isolated from the left cheek, whereas *Aspergillus versicolor* (Figure 2b) was isolated from the right calf, as shown in Table 1. Collection accession numbers, species identification, primers, and percentage identity values are provided in Table 2.

The number of publications analyzed over time is shown in Figure 3. Figure 4 and Figure 5 summarize the genera and phyla recorded from cadavers, respectively.

## 4. Discussion

### 4.1. Taxonomic Identification and Forensic Relevance of the Isolated Species

A multilocus identification approach for fungi, based on phylogenetic marker databases (ITS, BT, CMD, and LSU) and coupled with morphological observation, is considered a valid tool for fungal species identification [47]. In the present study, this approach allowed for the identification of four isolates collected from a cadaver: *Penicillium brevicompactum, Penicillium chrysogenum, Aspergillus versicolor, and Mucor saturninus*, as summarized in Table 2. The occurrence of these species is discussed below in relation to the available literature.

*Penicillium brevicompactum* was reported by Piepenbring et al. [7] on the lower lip of a cadaver in the decay stage. Other authors found it on two different bodies in the putrefaction stage [12]. Notably, Hawksworth and Wiltshire [11] reported the same species growing on a blood-wetted carpet. In the present study, *P. brevicompactum* was found on the left hand of a cadaver in the putrefaction stage.

Piepenbring et al. [7] reported *Penicillium chrysogenum* on three cadavers at two different decomposition stages. In a fresh cadaver, it was found on the thigh, whereas in a decay/partially mummified cadaver it was found in the genital area and the buccal cavity. The same species was also found in mummified human remains [45] and in the inguinal region of a fresh cadaver [46]. In the present study, *P. chrysogenum* was found on the left cheek of a cadaver in the putrefaction stage.

According to the available literature, *Aspergillus* spp. have been found on cadavers at different decomposition stages [2,5,7,12,48]. However, *Aspergillus versicolor* has previously been reported mainly from mummified human remains [45]. In the present study, *A. versicolor* was found on the right calf of a cadaver in the putrefaction stage; based on the literature consulted, this may represent the first report of this species from putrefactive human remains.

To the best of our knowledge, *Mucor saturninus* has previously been reported only by Schwarz et al. [12] in a putrefaction stage case. In the present study, *M. saturninus* was also found on the left cheek of a cadaver in the putrefaction stage.

### 4.2. Geographic and Taxonomic Overview of the Updated Checklist

Studies on cadaver-associated fungi have been reported from a relatively small number of countries, including Brazil, Croatia, Germany, Greenland, the Netherlands, Iraq, Italy, Japan, Poland, Switzerland, Thailand, and the United Kingdom. The earliest tradition of thanatomycology appears to have been established in Italy by Bianchini (1923) [27] and continued by Bianchini and Manfrini (1924) [28], Bianchini (1925) [29], and Ciferri et al. (1943) [30]. However, the number of listed culturable fungi remains relatively small, leaving several research gaps to be addressed [2,4,5,6,7,11,12,15,16,18,19,22,45,46,47,48,49,50,51]. Building on the dataset reported by Piepenbring et al. [7], and integrating newly recovered historical records and the present isolates, we observed that *Penicillium* spp. represented the most frequently reported genus isolated from human cadavers (23.6%), followed by *Yarrowia* spp. (10.2%), *Aspergillus* spp. (8.6%), *Mucor* spp. (7.1%), *Scopulariopsis* spp. (3.7%), and *Debaryomyces* spp. (3.5%), whereas all other genera collectively accounted for 43.3% of the isolates.

With regards to phyla, *Ascomycota* accounted for 86.2% of isolates, followed by *Mucoromycota* (9.6%) and *Basidiomycota* (3.3%), whereas *Mortierellomycota*, *Oomycota*, and *Dictyosteliomycota* each accounted for less than 1%. During the compilation of the checklist, several taxon names were updated to their currently accepted MycoBank nomenclature and numerous additional records were incorporated relative to previous compilations, as detailed in Table 3.

As shown in Figure 6, most research on fungi associated with human bodies has been conducted in Germany, Italy, and Poland, as also supported by Figure 7, which shows the diversity of genera collected in these countries. It is important to emphasize that fungi can be relevant in forensic investigations because they may help determine the locality and possible postmortem transfer of a body, estimate the postmortem interval through colonization time and growth rates of specific species, and indicate decomposition stages under stable indoor conditions based on their moisture requirements. In addition, fungi may contribute to assessing health risks for personnel, improving corpse preservation in medical and museum contexts, and supporting the development of environmentally sustainable burial practices [7]. It must be stressed that this geographic pattern is an artefact of uneven research intensity rather than evidence of a true biological or ecological trend: 53% of all checklist records originate from Poland alone, reflecting the large series of isolates recently reported by Spychała et al. [15] and their associated dataset, while Germany (21%) and Italy (15%) together account for most of the remaining records. Countries appearing at the bottom of the ranking have simply been the subject of very few forensic mycology studies to date, and not fewer cadaver-associated fungi. Consequently, the percentages reported in Figure 6 and Figure 7 should be interpreted as a map of research effort to date rather than as a representative picture of the global distribution of cadaver-associated fungi, and any biogeographic inference drawn from this checklist should be treated with corresponding caution. These gaps, together with the pronounced geographic and taxonomic unevenness of the existing literature, indicate that further systematic, culture-based surveys of cadaver-associated fungi are needed across a wider range of countries, climates, and decomposition contexts, in order to build a more representative and forensically useful picture of this fungal community.

### 4.3. Methodological Limitations

This study is based on the analysis of a single cadaver using culture-dependent isolation, an approach that inherently recovers only a fraction of the fungal community actually present on a decomposing body. Culture-independent, sequencing-based approaches have revealed considerably richer and more complex necrobiome assemblages than culture-dependent surveys typically detect. Burcham et al. [50], analyzing 36 human cadavers across three geographic locations using metagenomics and metabolomics, documented a phylogenetically conserved, interdomain microbial network that assembled during decomposition despite differences in location, climate, and season. Similarly, Fu et al. [51] characterized fungal succession patterns in decomposing pig carcasses using DNA metabarcoding, reporting distinct diversity trends between indoor and outdoor decomposition environments. More recently, Procopio et al. [52] applied ITS metabarcoding to simulated forensic burial contexts, showing that soil fungal community shifts can track the post-burial interval even when the carcass is not directly exposed, further underscoring the complementary value of culture-independent fungal profiling. Although culture-dependent methods cannot capture this full diversity, they remain indispensable for obtaining viable, taxonomically verified isolates that can be deposited in reference culture collections, a resource that sequencing-based approaches alone do not provide. Future studies combining both approaches across multiple cadavers and environmental settings would help reconcile the diversity recoverable by culturing with the broader necrobiome revealed by culture-independent techniques. In addition, no on-site measurements of relative humidity, temperature, or seasonal variation were available for the basement where the cadaver was found; only general outdoor data recorded at the Genoa Sestri Ponente weather station over the estimated PMI window (Section 2.1) could be retrieved, and these only approximate the conditions actually experienced by the body. Because such microclimatic parameters strongly influence fungal growth and sporulation, their absence limits direct comparison with other case reports and should be addressed in future forensic mycology protocols through on-site environmental logging. Indeed, Iancu et al. [53] showed that temperature and other environmental parameters are among the most informative predictors in microbiome-based PMI prediction models, reinforcing the importance of systematically recording such variables in future forensic mycology case studies. Similarly, although the cause of death was attributed to natural causes, the advanced putrefaction prevented a detailed anatomopathological examination and the postmortem interval could only be estimated broadly (approximately two weeks to three months) from the morphological findings at examination; this wide range, which could not be independently validated, limits the direct forensic interpretability of the fungal isolations reported here, and future cases should aim to couple fungal sampling with a narrower, medico-legally validated PMI estimate whenever possible.

## 5. Conclusions

Forensic mycological collections represent strategic ex situ repositories for the preservation, traceability, and future reuse of fungal strains isolated from forensic contexts. Dedicated forensic subsets can support taxonomic comparison, protocol standardization, and the development of reliable reference resources for forensic mycology. In this study, the deposition of *Penicillium brevicompactum*, *Penicillium chrysogenum*, *Mucor saturninus*, and *Aspergillus versicolor* in ColD UNIGE JRU MIRRI-IT strengthens the availability of reference strains for future comparative studies. The recovery of *A. versicolor* from a cadaver in the putrefaction stage expands current knowledge of culturable fungi associated with human decomposition and highlights the need for further research on fungal succession, anatomical sampling sites, and environmental drivers of cadaver-associated mycobiota. Such efforts may improve the interpretation of fungal evidence in relation to environmental conditions, postmortem interval estimation, and potential body movement. More broadly, the geographic and taxonomic gaps revealed by the updated checklist indicate that additional culture-based surveys of cadaver-associated fungi, conducted across a wider range of countries and decomposition settings, are needed to build a more complete and representative picture of this fungal community.

## Figures and Tables

**Table 1:** Samples, isolate identification, sampling location, culture medium, and sequence accession number.

Samples	Col.D Accession Number	Sampling Location	Substrate Used	GenBank Sequence Accession Numbers
1	027-D2	Right calf	SB-RB	ITS: PZ675951; CMD: PZ707412; LSU: PZ675957
2	026-E2	Left hand	SB-RB	ITS: PZ675949; LSU: PZ675955
3	026-E3	Left cheek	SB-RB	ITS: PZ675950; BT: PZ707411
4	026-E4	Left cheek	SB-RB	LSU: PZ675956

(SB) Sabouraud and (RB) Rose Bengal.

**Table 2:** Collection accession numbers, species identification, primers, and percentage identity values.

Col.D Accession Number ^(1)^	Species	- GenBank Sequence Accession Number Comparison - Amplicon Length - Percentage of Identity with First BLAST Result			
		ITS	CMD	BT	LSU
027-D2	*Aspergillus versicolor* (Vuill.) Tirab.	MZ422914.1 569 bps 100%	OP650535.1 697 bps 100%	-	MH876758.1 908 99.78%
026-E2	*Penicillium brevicompactum* Dierckx	MH047201.1 436 bps 100%	*-*	*-*	MH393340.1 1187 bps 100%
026-E3	*Penicillium chrysogenum* Thom	PX685536.1 505 bps 100%	-	PV057707.1 435 bps 100%	-
026-E4	*Mucor saturninus* Hagem.	-	-	-	MH870987.1 1025 bps 99.90%

^(1)^ Accession Nos. in the Collection of DISTAV. The GenBank accession numbers, sequence lengths, and percentage identity values reported in this table refer to the closest BLAST reference match for each marker, not to the sequences generated in this study. The accession numbers of the sequences deposited by the authors for each isolate and marker are reported in Table 1. ITS: internal transcribed spacer; CMD: partial calmodulin gene; BT: partial beta-tubulin gene; LSU: partial large subunit ribosomal RNA gene.

**Table 3:** The updated checklist of cadaver-associated culturable fungi (1923–2026), compiled from 27 literature sources across 12 countries together with the 4 isolates reported here (883 records in total). Taxon names follow current MycoBank nomenclature where verified; a hyphen (-) indicates data not reported in the original source. PMI = postmortem interval. Literature sources are listed in references [2,5,6,7,11,12,15,27,28,29,30,31,32,33,34,35,36,37,38,39,40,41,42,43,44,45,46].

Phylum	Species	Country	Locality	Decomposition Stage	PMI	Reference
-	*Glenosphora graphii* (Harz. Et Bez.) Vuill.	Italy	outdoors, buried	mummification/skeletonization	not indicated	Bianchini e Manfrini 1924
Ascomycota	*Acaulium album*	Poland	outdoors, close to water	decay/mummification/skeletonization	approximately 4 months	Spychała et al. 2026 a
Ascomycota	*Acaulium album*	Poland	outdoors, close to water	decay/mummification/skeletonization	approximately 4 months	Spychała et al. 2026 a
Ascomycota	*Acaulium album*	Poland	outdoors, close to water	decay/mummification/skeletonization	approximately 4 months	Spychała et al. 2026 a
Ascomycota	*Acaulium album*	Poland	outdoors, close to water	decay/mummification/skeletonization	approximately 4 months	Spychała et al. 2026 a
Ascomycota	*Acaulium album* (Costantin) Seifert & Woudenb.	Poland	outdoors	Fresh stage	-	Spychała et al. 2026 b
Ascomycota	*Acaulium album* (Costantin) Seifert & Woudenb.	Poland	outdoors	Fresh stage	-	Spychała et al. 2026 b
Ascomycota	*Acaulium caviariforme*	Poland	outdoors, close to water	decay/mummification/skeletonization	approximately 4 months	Spychała et al. 2026 a
Ascomycota	*Acaulium caviariforme*	Poland	outdoors, close to water	decay/mummification/skeletonization	approximately 4 months	Spychała et al. 2026 a
Ascomycota	*Acaulium caviariforme*	Poland	outdoors, close to water	decay/mummification/skeletonization	approximately 4 months	Spychała et al. 2026 a
Ascomycota	*Acaulium caviariforme*	Poland	outdoors, close to water	decay/mummification/skeletonization	approximately 4 months	Spychała et al. 2026 a
Ascomycota	*Acaulium caviariforme*	Poland	outdoors, close to water	decay/mummification/skeletonization	approximately 4 months	Spychała et al. 2026 a
Ascomycota	*Acaulium caviariforme*	Poland	outdoors, close to water	decay/mummification/skeletonization	approximately 4 months	Spychała et al. 2026 a
Ascomycota	*Acaulium caviariforme* (Malloch & Hubart) Sand.-Den., Guarro & Gené	Poland	outdoors	Fresh stage	-	Spychała et al. 2026 b
Ascomycota	*Acontium album* Morg.	Italy	outdoors, buried	mummification/skeletonization	not indicated	Ciferri R 1943
Ascomycota	*Acontium album* Morg.	Italy	outdoors, buried	mummification/skeletonization	not indicated	Ciferri R 1943
Ascomycota	*Acontium album* Morg.	Italy	outdoors, buried	mummification/skeletonization	not indicated	Ciferri R 1943
Ascomycota	*Acontium album* Morg.	Italy	outdoors, buried	mummification/skeletonization	not indicated	Ciferri R 1943
Ascomycota	*Acremoniella vaccini* (Fuck.) Sacc.	Italy	outdoors, buried	mummification/skeletonization	not indicated	Ciferri R 1943
Ascomycota	*Acremoniella vaccini* (Fuck.) Sacc.	Italy	outdoors, buried	mummification/skeletonization	not indicated	Ciferri R 1943
Ascomycota	*Acremonium bonordeni* Sacc.	Italy	outdoors, buried	mummification/skeletonization	not indicated	Ciferri R 1943
Ascomycota	*Acremonium* sp.	Brazil	indoors	fresh	not indicated	Sidrim et al. 2010
Ascomycota	*Acremonium* sp.	Brazil	indoors	fresh	not indicated	Sidrim et al. 2010
Ascomycota	*Acremonium* sp.	Brazil	-	advanced decay	not indicated	Sidrim et al. 2010
Ascomycota	*Acrocylindrium granulosum* Bonord.	Italy	outdoors, buried	mummification/skeletonization	not indicated	Ciferri R 1943
Ascomycota	*Acrostalagmus luteoalbus* (Link) Zare, W. Gams & Schroers	Italy	outdoors, buried	mummification/skeletonization	not indicated	Bianchini e Manfrini 1924
Ascomycota	*Akanthomyces lecanii*	Poland	outdoors, close to water	decay/mummification/skeletonization	approximately 4 months	Spychała et al. 2026 a
Ascomycota	*Alternaria alternata* (Fr.) Keissl.	Poland	outdoors	Advanced postmortem changes	-	Spychała et al. 2026 b
Ascomycota	*Alternaria infectoria* E.G. Simmons	Poland	outdoors	Decay with signs of mummification	-	Spychała et al. 2026 b
Ascomycota	*Amauroascus aureus* (Eidam) Arx	Italy	-	mummification/skeletonization	not indicated	Bianchini, G 1923
Ascomycota	*Aposphaeriopsis fuscoatra* var. hemisphaerica Cif. Et Red.	Italy	outdoors, buried	mummification/skeletonization	not indicated	Ciferri R 1943
Ascomycota	*Arachniotus aureus* (Eidam) Schröt.	Italy	outdoors, buried	mummification/skeletonization	not indicated	Bianchini e Manfrini 1924
Ascomycota	*Arthrographis kalrae* (R.P. Tewari & Macph.) Sigler & J.W. Carmich.	Poland	outdoors	Advanced decay	-	Spychała et al. 2026 b
Ascomycota	*Ascospirella lutea* (Zukal) Houbraken, Frisvad & Samson	Germany	outdoors, buried	not indicated	2 years	Dósa 1955
Ascomycota	*Ascospirella lutea* (Zukal) Houbraken, Frisvad & Samson	Germany	outdoors, buried	not indicated	2 years	Dósa 1955
Ascomycota	*Ascospirella lutea* (Zukal) Houbraken, Frisvad & Samson	Germany	outdoors, buried	not indicated	2 years	Dósa 1955
Ascomycota	*Ascospirella lutea* (Zukal) Houbraken, Frisvad & Samson	Germany	outdoors, buried	not indicated	2 years	Dósa 1955
Ascomycota	*Ascospirella lutea* (Zukal) Houbraken, Frisvad & Samson	Germany	outdoors, buried	not indicated	2 years	Dósa 1955
Ascomycota	*Ascospirella lutea* (Zukal) Houbraken, Frisvad & Samson	Germany	outdoors, buried	not indicated	2 years	Dósa 1955
Ascomycota	*Aspergillus*	Iraq	indoors	not indicated	not indicated (long)	Hammad et al. 2002
Ascomycota	*Aspergillus*	Italy	indoors	mummification/skeletonization	more than 100 years	Piñar et al. 2013
Ascomycota	*Aspergillus candidus* (Pers.) Link	Italy	-	advanced decay	not indicated	Bianchini, G 1923
Ascomycota	*Aspergillus candidus* Link	Germany	outdoors, buried	not indicated	4 years	Dósa 1955
Ascomycota	*Aspergillus candidus* Link	Germany	outdoors, buried	not indicated	2 years	Dósa 1955
Ascomycota	*Aspergillus candidus* Link	Germany	outdoors, buried	not indicated	3 years	Dósa 1955
Ascomycota	*Aspergillus candidus* Link	Italy	outdoors, buried	advanced decay	not indicated	Bianchini e Manfrini 1924
Ascomycota	*Aspergillus chevalieri* (L. Mangin) Thom & Church	Japan	outdoors	mummification/skeletonization	>6 months	Ishii et al. 2006
Ascomycota	*Aspergillus clavatus* Desm.	Germany	outdoors, buried	not indicated	3 days	Dósa 1955
Ascomycota	*Aspergillus clavatus* Desm.	Germany	outdoors, buried	not indicated	2 months	Dósa 1955
Ascomycota	*Aspergillus clavatus* Desm.	Germany	outdoors, buried	not indicated	2 weeks	Dósa 1955
Ascomycota	*Aspergillus clavatus* Desm.	Germany	outdoors, buried	not indicated	3 months	Dósa 1955
Ascomycota	*Aspergillus flavus* Link	Brazil	-	advanced decay	not indicated	Sidrim et al. 2010
Ascomycota	*Aspergillus flavus* Link	Brazil	-	advanced decay	not indicated	Sidrim et al. 2010
Ascomycota	*Aspergillus flavus* Link	Brazil	indoors	fresh	not indicated	Sidrim et al. 2010
Ascomycota	*Aspergillus flavus* Link	Brazil	indoors	fresh	not indicated	Sidrim et al. 2010
Ascomycota	*Aspergillus flavus* Link	Brazil	indoors	fresh	not indicated	Sidrim et al. 2010
Ascomycota	*Aspergillus flavus* Link	Brazil	outdoors	mummification/skeletonization	not indicated	Sidrim et al. 2010
Ascomycota	*Aspergillus flavus* Link	Brazil	indoors	fresh	not indicated	Sidrim et al. 2010
Ascomycota	*Aspergillus flavus* Link	Germany	outdoors, buried	not indicated	2 years	Dósa 1955
Ascomycota	*Aspergillus flavus* Link	Germany	outdoors, buried	not indicated	2 years	Dósa 1955
Ascomycota	*Aspergillus flavus* Link	Germany	outdoors, buried	not indicated	2 years	Dósa 1955
Ascomycota	*Aspergillus flavus* Link	Germany	outdoors, buried	not indicated	2 years	Dósa 1955
Ascomycota	*Aspergillus flavus* Link	Germany	outdoors, buried	not indicated	2 years	Dósa 1955
Ascomycota	*Aspergillus flavus* Link	Germany	outdoors, buried	not indicated	2 years	Dósa 1955
Ascomycota	*Aspergillus flavus* Link	Germany	outdoors, buried	not indicated	2 years	Dósa 1955
Ascomycota	*Aspergillus flavus* Link	Germany	outdoors, buried	not indicated	2 years	Dósa 1955
Ascomycota	*Aspergillus flavus* Link	Germany	indoors	advanced decay	4–6 days	Schwarz et al. 2015
Ascomycota	*Aspergillus flavus* Link	Poland	outdoors	decay with signs of mummification	-	Spychała et al. 2026 b
Ascomycota	*Aspergillus fumigatus* Fresen.	Croatia	indoors	mummification/skeletonization	archaeological	Čavka et al. 2010
Ascomycota	*Aspergillus fumigatus* Fresen.	Germany	outdoors, buried	not indicated	2 years	Dósa 1955
Ascomycota	*Aspergillus fumigatus* Fresen.	Germany	outdoors, buried	not indicated	2 years	Dósa 1955
Ascomycota	*Aspergillus fumigatus* Fresen.	Germany	outdoors, buried	not indicated	2 years	Dósa 1955
Ascomycota	*Aspergillus fumigatus* Fresen.	Germany	outdoors, buried	not indicated	2 years	Dósa 1955
Ascomycota	*Aspergillus fumigatus* Fresen.	Germany	outdoors, buried	not indicated	2 years	Dósa 1955
Ascomycota	*Aspergillus fumigatus* Fresen.	Germany	indoors	advanced decay	not indicated	Schwarz et al. 2015
Ascomycota	*Aspergillus fumigatus* Fresen.	Germany	outdoors, buried	decay/mummification/skeletonization	5 weeks	Schwarz et al. 2015
Ascomycota	*Aspergillus fumigatus* Fresen.	Germany	outdoors	decay/mummification/skeletonization	not indicated	Schwarz et al. 2015
Ascomycota	*Aspergillus fumigatus* Fresen.	Italy	-	not indicated	-	Bianchini, G (1925) 1925
Ascomycota	*Aspergillus fumigatus* Fresen.	Poland	outdoors	advanced decay	not indicated	Spychała, K 2025
Ascomycota	*Aspergillus glaucus* (L.) Fr.	Italy	outdoors, buried	fresh/decay	not indicated	Bianchini e Manfrini 1924
Ascomycota	*Aspergillus glaucus* (L.) Fr.	Italy	outdoors, buried	advanced decay	not indicated	Bianchini e Manfrini 1924
Ascomycota	*Aspergillus glaucus* (L.) Fr.	Italy	outdoors, buried	advanced decay	not indicated	Ciferri R 1943
Ascomycota	*Aspergillus glaucus* (L.) Fr.	Italy	-	advanced decay	not indicated	Bianchini, G 1923
Ascomycota	*Aspergillus glaucus* (L.) Link	Italy	-	fresh/decay	not indicated	Bianchini, G 1923
Ascomycota	*Aspergillus nidulans* (Eidam) G. Winter	Croatia	indoors	mummification/skeletonization	archaeological	Čavka et al. 2010
Ascomycota	*Aspergillus niger*	Poland	outdoors, close to water	decay/mummification/skeletonization	approximately 4 months	Spychała et al. 2026 a
Ascomycota	*Aspergillus niger* Tiegh.	Brazil	-	advanced decay	not indicated	Sidrim et al. 2010
Ascomycota	*Aspergillus niger* Tiegh.	Brazil	indoors	fresh	not indicated	Sidrim et al. 2010
Ascomycota	*Aspergillus niger* Tiegh.	Brazil	indoors	fresh	not indicated	Sidrim et al. 2010
Ascomycota	*Aspergillus niger* Tiegh.	Brazil	indoors	fresh	not indicated	Sidrim et al. 2010
Ascomycota	*Aspergillus niger* Tiegh.	Brazil	indoors	fresh	not indicated	Sidrim et al. 2010
Ascomycota	*Aspergillus niger* Tiegh.	Brazil	outdoors	mummification/skeletonization	not indicated	Sidrim et al. 2010
Ascomycota	*Aspergillus niger* Tiegh.	Germany	outdoors, buried	not indicated	10 years	Dósa 1955
Ascomycota	*Aspergillus niger* Tiegh.	Germany	indoors	decay/mummification/skeletonization	79 days	Piepenbring, M 2025
Ascomycota	*Aspergillus niger* Tiegh.	Italy	outdoors, buried	mummification/skeletonization	not indicated	Bianchini e Manfrini 1924
Ascomycota	*Aspergillus niger* Tiegh.	Poland	outdoors	fresh stage	-	Spychała et al. 2026 b
Ascomycota	*Aspergillus niger* Tiegh.	Poland	outdoors	fresh stage	-	Spychała et al. 2026 b
Ascomycota	*Aspergillus niger* Tiegh.	Poland	outdoors	decay with signs of mummification	-	Spychała et al. 2026 b
Ascomycota	*Aspergillus niger* Tiegh.	Poland	outdoors	decay with signs of mummification	-	Spychała et al. 2026 b
Ascomycota	*Aspergillus niger* Tiegh.	Poland	outdoors	decay with signs of mummification	-	Spychała et al. 2026 b
Ascomycota	*Aspergillus niger* Tiegh.	Poland	outdoors	decay with signs of mummification	-	Spychała et al. 2026 b
Ascomycota	*Aspergillus niger* Tiegh.	Poland	outdoors	decay with signs of mummification	-	Spychała et al. 2026 b
Ascomycota	*Aspergillus niger* Tiegh.	Poland	outdoors	decay with signs of mummification	-	Spychała et al. 2026 b
Ascomycota	*Aspergillus niger* Tiegh.	Poland	outdoors	decay with signs of mummification	-	Spychała et al. 2026 b
Ascomycota	*Aspergillus niger* Tiegh.	Poland	outdoors	decay with signs of mummification	-	Spychała et al. 2026 b
Ascomycota	*Aspergillus ochraceus* Wilh.	Italy	outdoors, buried	advanced decay	not indicated	Ciferri R 1943
Ascomycota	*Aspergillus pseudoglaucus* Blochwitz	Germany	indoors	decay/mummification/skeletonization	not indicated	Schwarz et al. 2015
Ascomycota	*Aspergillus pseudoglaucus* Blochwitz	Germany	outdoors	decay/mummification/skeletonization	not indicated	Schwarz et al. 2015
Ascomycota	*Aspergillus pseudoglaucus* Blochwitz	Japan	indoors	mummification/skeletonization	10 months	Ishii et al. 2006
Ascomycota	*Aspergillus pseudoglaucus* Blochwitz	Japan	outdoors	mummification/skeletonization	>6 months	Ishii et al. 2006
Ascomycota	*Aspergillus terreus* Thom	Japan	outdoors	decay/mummification/skeletonization	not indicated	Hitosugi et al. 2006
Ascomycota	*Aspergillus vadensis* Samson, R.P. de Vries, Frisvad & Visser	Poland	outdoors	advanced decay	-	Spychała et al. 2026 b
Ascomycota	*Aspergillus versicolor* “(Vuill.) Tirab”	Italy	indoors	advanced decay	-	Iannaccone et al. 2026 current
Ascomycota	*Aspergillus versicolor* “(Vuill.) Tirab”	Poland	outdoors	advanced decay	-	Spychała et al. 2026 b
Ascomycota	*Aspergillus versicolor* “(Vuill.) Tirab”	Poland	outdoors	advanced decay	-	Spychała et al. 2026 b
Ascomycota	*Biscogniauxia mediterranea* (De Not.) Kuntze	Germany	outdoors	advanced decay	11 days	Piepenbring, M 2025
Ascomycota	*Biscogniauxia mediterranea* (De Not.) Kuntze	Germany	outdoors, water	advanced decay	>6 days	Piepenbring, M 2025
Ascomycota	*Blastobotrys fungorum* (de Hoog & G.A. de Vries) Visagie, Seifert & Boekhout	Greenland	outdoors, buried	mummification/skeletonization	500 years	Bodenhoff et al. 1979
Ascomycota	*Botryobasidium capitatum* (Link) Rossman & W.C. Allen	Italy	outdoors, buried	mummification/skeletonization	not indicated	Ciferri R 1943
Ascomycota	*Botryotrichum domesticum* D.W. Li & N.P. Schultes,	Poland	outdoors	advanced decay	not indicated	Spychała, K 2025
Ascomycota	*Candida albicans* (C.P. Robin) Berkhout	Brazil	-	advanced decay	not indicated	Sidrim et al. 2010
Ascomycota	*Candida albicans* (C.P. Robin) Berkhout	Brazil	-	advanced decay	not indicated	Sidrim et al. 2010
Ascomycota	*Candida albicans* (C.P. Robin) Berkhout	Brazil	indoors	fresh	not indicated	Sidrim et al. 2010
Ascomycota	*Candida albicans* (C.P. Robin) Berkhout	Brazil	indoors	fresh	not indicated	Sidrim et al. 2010
Ascomycota	*Candida albicans* (C.P. Robin) Berkhout	Brazil	-	advanced decay	not indicated	Sidrim et al. 2010
Ascomycota	*Candida albicans* (C.P. Robin) Berkhout	Brazil	indoors	fresh	not indicated	Sidrim et al. 2010
Ascomycota	*Candida albicans* (C.P. Robin) Berkhout	Brazil	indoors	fresh	not indicated	Sidrim et al. 2010
Ascomycota	*Candida albicans* (C.P. Robin) Berkhout	Germany	indoors	advanced decay	not indicated	Schwarz et al. 2015
Ascomycota	*Candida albicans* (C.P. Robin) Berkhout	Poland	outdoors	advanced decay	not indicated	Spychała, K 2025
Ascomycota	*Candida tropicalis* (Castell.) Berkhout	Germany	-	advanced decay	not indicated	Schwarz et al. 2015
Ascomycota	*Cephalosporium acremonium* Corda	Italy	outdoors, buried	mummification/skeletonization	not indicated	Ciferri R 1943
Ascomycota	*Cephalosporium acremonium* Corda	Italy	outdoors, buried	mummification/skeletonization	not indicated	Ciferri R 1943
Ascomycota	*Cephalosporium acremonium* Corda	Italy	outdoors, buried	mummification/skeletonization	not indicated	Ciferri R 1943
Ascomycota	*Cephalotrichum stemonitis* (Pers.) Nees	Switzerland	outdoors, buried	mummification/skeletonization	more than 200 years	Piepenbrink 1986
Ascomycota	*Chaetomium globosum* Kunze	Germany	outdoors, buried	not indicated	10 years	Dósa 1955
Ascomycota	*Chaetomium subaffine* Sergeeva	Poland	outdoors	advanced decay	not indicated	Spychała, K 2025
Ascomycota	*Chrysosporium* spp.	Croatia	indoors	mummification/skeletonization	archaeological	Čavka et al. 2010
Ascomycota	*Cladosporium allicinum* (Fr.) Bensch, U. Braun & Crous	Poland	outdoors	fresh stage	-	Spychała et al. 2026 b
Ascomycota	*Cladosporium allicinum* (Fr.) Bensch, U. Braun & Crous	Poland	outdoors	fresh stage	-	Spychała et al. 2026 b
Ascomycota	*Cladosporium cladosporioides* (Fresen.) G.A. de Vries	Germany	indoors	fresh/decay	22 days	Piepenbring, M 2025
Ascomycota	*Cladosporium cladosporioides* (Fresen.) G.A. de Vries	Italy	indoors	not indicated	21 days	Di Piazza et al. 2018
Ascomycota	*Cladosporium cladosporioides* (Fresen.) G.A. de Vries	Japan	outdoors	advanced decay	4 months	Kato et al. 2023
Ascomycota	*Cladosporium cladosporioides* (Fresen.) G.A. de Vries	Poland	outdoors	fresh stage	-	Spychała et al. 2026 b
Ascomycota	*Cladosporium cladosporioides* (Fresen.) G.A. de Vries	Poland	outdoors	fresh stage	-	Spychała et al. 2026 b
Ascomycota	*Cladosporium colocasiae* Sawada	Thailand	indoors	not indicated	not indicated (long)	Sri-Indrasutdhi et al. 2015
Ascomycota	*Cladosporium cucumerinum* Ellis & Arthur	Germany	indoors	fresh	11 days	Piepenbring, M 2025
Ascomycota	*Cladosporium halotolerans* Zalar, de Hoog & Gunde-Cim.	Poland	outdoors	Fresh stage	-	Spychała et al. 2026 b
Ascomycota	*Cladosporium herbarum* (Pers.) Link	Poland	outdoors	decay with signs of mummification	-	Spychała et al. 2026 b
Ascomycota	*Cladosporium langeronii* (Fonseca, Leão & Nogueira) Vuill.	Poland	outdoors	fresh stage	-	Spychała et al. 2026 b
Ascomycota	*Cladosporium* sp.	Holland	indoors	not indicated	several weeks	van de Voorde & van Dijck 1982
Ascomycota	*Cladosporium* sp.	Japan	outdoors	advanced decay	1 month	Kato et al. 2023
Ascomycota	*Clonostachys candelabrum* (Bonord.) Schroers	Italy	-	mummification/skeletonization	not indicated	Bianchini, G 1923
Ascomycota	*Clonostachys rosea* (Link) Schroers, Samuels, Seifert & W. Gams	Italy	outdoors, buried	mummification/skeletonization	not indicated	Ciferri R 1943
Ascomycota	*Collacystis putredinis* Kunze	Italy	outdoors, buried	mummification/skeletonization	not indicated	Bianchini e Manfrini 1924
Ascomycota	*Cosmospora khandalensis* (Thirum. & Sukapure) Gräfenhan & Seifert	Poland	outdoors	advanced decay	-	Spychała et al. 2026 b
Ascomycota	*Cosmospora khandalensis* (Thirum. & Sukapure) Gräfenhan & Seifert	Poland	outdoors	advanced decay	-	Spychała et al. 2026 b
Ascomycota	*Cosmospora khandalensis* (Thirum. & Sukapure) Gräfenhan & Seifert	Poland	outdoors	advanced decay	-	Spychała et al. 2026 b
Ascomycota	*Cosmospora khandalensis* (Thirum. & Sukapure) Gräfenhan & Seifert	Poland	outdoors	advanced decay	-	Spychała et al. 2026 b
Ascomycota	*Ctenomyces serratus* Eidam	Italy	outdoors, buried	advanced decay	not indicated	Bianchini e Manfrini 1924
Ascomycota	*Ctenomyces serratus* Eidam	Italy	-	advanced decay	not indicated	Bianchini, G 1923
Ascomycota	*Cylindrium elongatum* Bonord.	Italy	outdoors, buried	mummification/skeletonization	not indicated	Ciferri R 1943
Ascomycota	*Debaryomyces hansenii* (Zopf) Lodder & Kreger-van Rij	Germany	outdoors	decay/mummification/skeletonization	not indicated	Schwarz et al. 2015
Ascomycota	*Debaryomyces hansenii* (Zopf) Lodder & Kreger-van Rij	Poland	outdoors	advanced decay	not indicated	Spychała, K 2025
Ascomycota	*Debaryomyces hansenii* (Zopf) Lodder & Kreger-van Rij	Poland	outdoors	fresh stage	-	Spychała et al. 2026 b
Ascomycota	*Debaryomyces hansenii* (Zopf) Lodder & Kreger-van Rij	Poland	outdoors	advanced decay	-	Spychała et al. 2026 b
Ascomycota	*Debaryomyces hansenii* (Zopf) Lodder & Kreger-van Rij	Poland	outdoors	fresh stage	-	Spychała et al. 2026 b
Ascomycota	*Debaryomyces hansenii* (Zopf) Lodder & Kreger-van Rij	Poland	outdoors	fresh stage	-	Spychała et al. 2026 b
Ascomycota	*Debaryomyces hansenii* (Zopf) Lodder & Kreger-van Rij	Poland	outdoors	fresh stage	-	Spychała et al. 2026 b
Ascomycota	*Debaryomyces hansenii* (Zopf) Lodder & Kreger-van Rij	Poland	outdoors	fresh stage	-	Spychała et al. 2026 b
Ascomycota	*Debaryomyces hansenii* (Zopf) Lodder & Kreger-van Rij	Poland	outdoors	fresh stage	-	Spychała et al. 2026 b
Ascomycota	*Debaryomyces hansenii* (Zopf) Lodder & Kreger-van Rij	Poland	outdoors	fresh stage	-	Spychała et al. 2026 b
Ascomycota	*Debaryomyces hansenii* (Zopf) Lodder & Kreger-van Rij	Poland	outdoors	fresh stage	-	Spychała et al. 2026 b
Ascomycota	*Debaryomyces hansenii* (Zopf) Lodder & Kreger-van Rij	Poland	outdoors	advanced postmortem changes	-	Spychała et al. 2026 b
Ascomycota	*Debaryomyces hansenii* (Zopf) Lodder & Kreger-van Rij	Poland	outdoors	advanced postmortem changes	-	Spychała et al. 2026 b
Ascomycota	*Debaryomyces hansenii* (Zopf) Lodder & Kreger-van Rij	Poland	outdoors	advanced postmortem changes	-	Spychała et al. 2026 b
Ascomycota	*Debaryomyces hansenii* (Zopf) Lodder & Kreger-van Rij	Poland	outdoors	advanced postmortem changes	-	Spychała et al. 2026 b
Ascomycota	*Debaryomyces hansenii* (Zopf) Lodder & Kreger-van Rij	Poland	outdoors	advanced postmortem changes	-	Spychała et al. 2026 b
Ascomycota	*Debaryomyces hansenii* (Zopf) Lodder & Kreger-van Rij	Poland	outdoors	advanced postmortem changes	-	Spychała et al. 2026 b
Ascomycota	*Debaryomyces hansenii* (Zopf) Lodder & Kreger-van Rij	Poland	outdoors	advanced postmortem changes	-	Spychała et al. 2026 b
Ascomycota	*Debaryomyces hansenii* (Zopf) Lodder & Kreger-van Rij	Poland	outdoors	advanced postmortem changes	-	Spychała et al. 2026 b
Ascomycota	*Debaryomyces hansenii* (Zopf) Lodder & Kreger-van Rij	Poland	outdoors	advanced postmortem changes	-	Spychała et al. 2026 b
Ascomycota	*Debaryomyces hansenii* (Zopf) Lodder & Kreger-van Rij	Poland	outdoors	advanced postmortem changes	-	Spychała et al. 2026 b
Ascomycota	*Debaryomyces hansenii* (Zopf) Lodder & Kreger-van Rij	Poland	outdoors	decay with signs of mummification	-	Spychała et al. 2026 b
Ascomycota	*Debaryomyces hansenii* (Zopf) Lodder & Kreger-van Rij	Poland	outdoors	decay with signs of mummification	-	Spychała et al. 2026 b
Ascomycota	*Debaryomyces hansenii* (Zopf) Lodder & Kreger-van Rij	Poland	outdoors	decay with signs of mummification	-	Spychała et al. 2026 b
Ascomycota	*Debaryomyces hansenii* (Zopf) Lodder & Kreger-van Rij	Poland	outdoors	decay with signs of mummification	-	Spychała et al. 2026 b
Ascomycota	*Debaryomyces hansenii* (Zopf) Lodder & Kreger-van Rij	Poland	outdoors	decay with signs of mummification	-	Spychała et al. 2026 b
Ascomycota	*Debaryomyces hansenii* (Zopf) Lodder & Kreger-van Rij	Poland	outdoors	decay with signs of mummification	-	Spychała et al. 2026 b
Ascomycota	*Debaryomyces hansenii* (Zopf) Lodder & Kreger-van Rij	Poland	outdoors	decay with signs of mummification	-	Spychała et al. 2026 b
Ascomycota	*Debaryomyces hansenii* (Zopf) Lodder & Kreger-van Rij	Poland	outdoors	decay with signs of mummification	-	Spychała et al. 2026 b
Ascomycota	*Debaryomyces hansenii* (Zopf) Lodder & Kreger-van Rij	Poland	outdoors	decay with signs of mummification	-	Spychała et al. 2026 b
Ascomycota	*Debaryomyces hansenii* (Zopf) Lodder & Kreger-van Rij	Poland	outdoors	decay with signs of mummification	-	Spychała et al. 2026 b
Ascomycota	*Dendryphion comosum* Wallr.	Germany	outdoors, buried	not indicated	3 years	Dósa 1955
Ascomycota	*Dialonectria ullevolea*	Poland	outdoors, close to water	decay/mummification/skeletonization	approximately 4 months	Spychała et al. 2026 a
Ascomycota	*Dialonectria ullevolea*	Poland	outdoors, close to water	decay/mummification/skeletonization	approximately 4 months	Spychała et al. 2026 a
Ascomycota	*Dialonectria ullevolea*	Poland	outdoors, close to water	decay/mummification/skeletonization	approximately 4 months	Spychała et al. 2026 a
Ascomycota	*Dialonectria ullevolea*	Poland	outdoors, close to water	decay/mummification/skeletonization	approximately 4 months	Spychała et al. 2026 a
Ascomycota	*Dialonectria volutella* (Ellis & Everh.) L. Lombard & Sand.-Den.	Poland	outdoors	advanced decay	-	Spychała et al. 2026 b
Ascomycota	*Dialonectria volutella* (Ellis & Everh.) L. Lombard & Sand.-Den.	Poland	outdoors	advanced decay	-	Spychała et al. 2026 b
Ascomycota	*Dujonia zeylanoides* (Castell.) Q.M. Wang, Yurkov, Boekhout & F.Y. Bai, Fungal Diversity 136 (136006): 67 (2026)	Poland	outdoors	fresh stage	-	Spychała et al. 2026 b
Ascomycota	*Dujonia zeylanoides* (Castell.) Q.M. Wang, Yurkov, Boekhout & F.Y. Bai, Fungal Diversity 136 (136006): 67 (2026)	Poland	outdoors	advanced decay	-	Spychała et al. 2026 b
Ascomycota	*Dujonia zeylanoides* (Castell.) Q.M. Wang, Yurkov, Boekhout & F.Y. Bai, Fungal Diversity 136 (136006): 67 (2026)	Poland	outdoors	fresh stage	-	Spychała et al. 2026 b
Ascomycota	*Dujonia zeylanoides* (Castell.) Q.M. Wang, Yurkov, Boekhout & F.Y. Bai, Fungal Diversity 136 (136006): 67 (2026)	Poland	outdoors	fresh stage	-	Spychała et al. 2026 b
Ascomycota	*Dujonia zeylanoides* (Castell.) Q.M. Wang, Yurkov, Boekhout & F.Y. Bai, Fungal Diversity 136 (136006): 67 (2026)	Poland	outdoors	fresh stage	-	Spychała et al. 2026 b
Ascomycota	*Dujonia zeylanoides* (Castell.) Q.M. Wang, Yurkov, Boekhout & F.Y. Bai, Fungal Diversity 136 (136006): 67 (2026)	Poland	outdoors	fresh stage	-	Spychała et al. 2026 b
Ascomycota	*Dujonia zeylanoides* (Castell.) Q.M. Wang, Yurkov, Boekhout & F.Y. Bai, Fungal Diversity 136 (136006): 67 (2026)	Poland	outdoors	decay with signs of mummification	-	Spychała et al. 2026 b
Ascomycota	*Dujonia zeylanoides* (Castell.) Q.M. Wang, Yurkov, Boekhout & F.Y. Bai, Fungal Diversity 136 (136006): 67 (2026)	Poland	outdoors	decay with signs of mummification	-	Spychała et al. 2026 b
Ascomycota	*Dujonia zeylanoides* (Castell.) Q.M. Wang, Yurkov, Boekhout & F.Y. Bai, Fungal Diversity 136 (136006): 67 (2026)	Poland	outdoors	decay with signs of mummification	-	Spychała et al. 2026 b
Ascomycota	*Dujonia zeylanoides* (Castell.) Q.M. Wang, Yurkov, Boekhout & F.Y. Bai, Fungal Diversity 136 (136006): 67 (2026)	Poland	outdoors	decay with signs of mummification	-	Spychała et al. 2026 b
Ascomycota	*Epicoccum nigrum* Link	Germany	indoors	fresh/decay	22 days	Piepenbring, M 2025
Ascomycota	*Epicoccum nigrum* Link	Japan	outdoors	advanced decay	1 month	Kato et al. 2023
Ascomycota	*Epicoccum nigrum* Link	Poland	outdoors	fresh stage	-	Spychała et al. 2026 b
Ascomycota	*Epidermophyton* sp.	Iraq	indoors	not indicated	not indicated (long)	Hammad et al. 2002
Ascomycota	*Eupenicillium* sp.	Italy	indoors	mummification/skeletonization	more than 100 years	Piñar et al. 2013
Ascomycota	*Eupenidiella venezuelensis* (Crous & U. Braun) Quaedvl. & Crous	Italy	indoors	mummification/skeletonization	more than 100 years	Piñar et al. 2013
Ascomycota	*Eurotium rubrum* Jos. König et al.	Japan	indoors	mummification/skeletonization	10 months	Ishii et al. 2006
Ascomycota	*Fermentozyma sake* (Saito & M. Ota) Q.M. Wang, Yurkov, Boekhout & F.Y. Bai	Poland	outdoors	advanced postmortem changes	-	Spychała et al. 2026 b
Ascomycota	*Fumago* sp.	Germany	outdoors, buried	not indicated	11 years	Dósa 1955
Ascomycota	*Fusarium acuminatum*	Poland	outdoors, close to water	decay/mummification/skeletonization	approximately 4 months	Spychała et al. 2026 a
Ascomycota	*Fusarium acuminatum*	Poland	outdoors, close to water	decay/mummification/skeletonization	approximately 4 months	Spychała et al. 2026 a
Ascomycota	*Fusarium acuminatum* Ellis & Everh.	Poland	outdoors	fresh stage	-	Spychała et al. 2026 b
Ascomycota	*Fusarium avenaceum* (Fr.) Sacc.	Italy	outdoors, buried	mummification/skeletonization	not indicated	Bianchini e Manfrini 1924
Ascomycota	*Fusarium chlamydosporum* Wollenw. & Reinking	Italy	outdoors, buried	mummification/skeletonization	not indicated	Bianchini e Manfrini 1924
Ascomycota	*Fusarium chlamydosporum* Wollenw. Reinking	Italy	-	mummification/skeletonization	not indicated	Bianchini, G 1923
Ascomycota	*Fusarium equiseti* (Corda) Sacc.	Italy	outdoors, buried	mummification/skeletonization	not indicated	Bianchini e Manfrini 1924
Ascomycota	*Fusarium falsibabinda*	Poland	outdoors, close to water	decay/mummification/skeletonization	approximately 4 months	Spychała et al. 2026 a
Ascomycota	*Fusarium oxysporum*	Poland	outdoors, close to water	decay/mummification/skeletonization	approximately 4 months	Spychała et al. 2026 a
Ascomycota	*Fusarium poae* (Peck) Wollenw.	Poland	outdoors	advanced decay	not indicated	Spychała, K 2025
Ascomycota	*Fusarium redolens*	Poland	outdoors, close to water	decay/mummification/skeletonization	approximately 4 months	Spychała et al. 2026 a
Ascomycota	*Fusarium redolens*	Poland	outdoors, close to water	decay/mummification/skeletonization	approximately 4 months	Spychała et al. 2026 a
Ascomycota	*Fusarium sacchari*	Poland	outdoors, close to water	decay/mummification/skeletonization	approximately 4 months	Spychała et al. 2026 a
Ascomycota	*Fusarium sambucinum* Fuckel	Italy	outdoors, buried	mummification/skeletonization	not indicated	Bianchini e Manfrini 1924
Ascomycota	*Fusarium solani*	Poland	outdoors, close to water	decay/mummification/skeletonization	approximately 4 months	Spychała et al. 2026 a
Ascomycota	*Fusarium solani*	Poland	outdoors, close to water	decay/mummification/skeletonization	approximately 4 months	Spychała et al. 2026 a
Ascomycota	*Fusarium solani*	Poland	outdoors, close to water	decay/mummification/skeletonization	approximately 4 months	Spychała et al. 2026 a
Ascomycota	*Fusarium solani*	Poland	outdoors, close to water	decay/mummification/skeletonization	approximately 4 months	Spychała et al. 2026 a
Ascomycota	*Fusarium solani*	Poland	outdoors, close to water	decay/mummification/skeletonization	approximately 4 months	Spychała et al. 2026 a
Ascomycota	*Fusarium* sp.	Brazil	indoors	fresh	not indicated	Sidrim et al. 2010
Ascomycota	*Fusarium* sp.	Holland	indoors	not indicated	several weeks	van de Voorde & van Dijck 1982
Ascomycota	*Fusarium* sp.	UK	outdoors, water	not indicated	at least 5 weeks in water	Hawksworth & Wiltshire 2011
Ascomycota	*Fusicolla aquaeductuum*	Poland	outdoors, close to water	decay/mummification/skeletonization	approximately 4 months	Spychała et al. 2026 a
Ascomycota	*Fusicolla aquaeductuum*	Poland	outdoors, close to water	decay/mummification/skeletonization	approximately 4 months	Spychała et al. 2026 a
Ascomycota	*Fusicolla aquaeductuum*	Poland	outdoors, close to water	decay/mummification/skeletonization	approximately 4 months	Spychała et al. 2026 a
Ascomycota	*Fusicolla aquaeductuum* (Radlk. & Rabenh.) Gräfenhan, Seifert & Schroers	Poland	outdoors	advanced decay	-	Spychała et al. 2026 b
Ascomycota	*Geotrichum candidum*	Poland	outdoors, close to water	decay/mummification/skeletonization	approximately 4 months	Spychała et al. 2026 a
Ascomycota	*Geotrichum candidum*	Poland	outdoors, close to water	decay/mummification/skeletonization	approximately 4 months	Spychała et al. 2026 a
Ascomycota	*Geotrichum candidum*	Poland	outdoors, close to water	decay/mummification/skeletonization	approximately 4 months	Spychała et al. 2026 a
Ascomycota	*Geotrichum candidum*	Poland	outdoors, close to water	decay/mummification/skeletonization	approximately 4 months	Spychała et al. 2026 a
Ascomycota	*Geotrichum candidum*	Poland	outdoors, close to water	decay/mummification/skeletonization	approximately 4 months	Spychała et al. 2026 a
Ascomycota	*Geotrichum candidum*	Poland	outdoors, close to water	decay/mummification/skeletonization	approximately 4 months	Spychała et al. 2026 a
Ascomycota	*Geotrichum candidum*	Poland	outdoors, close to water	decay/mummification/skeletonization	approximately 4 months	Spychała et al. 2026 a
Ascomycota	*Geotrichum candidum*	Poland	outdoors, close to water	decay/mummification/skeletonization	approximately 4 months	Spychała et al. 2026 a
Ascomycota	*Geotrichum candidum*	Poland	outdoors, close to water	decay/mummification/skeletonization	approximately 4 months	Spychała et al. 2026 a
Ascomycota	*Geotrichum candidum* Link	Germany	indoors	fresh	9 days	Piepenbring, M 2025
Ascomycota	*Geotrichum candidum* Link	Germany	indoors	advanced decay	20 days	Piepenbring, M 2025
Ascomycota	*Geotrichum candidum* Link	Holland	indoors	not indicated	several weeks	van de Voorde & van Dijck 1982
Ascomycota	*Geotrichum candidum* Link	Italy	outdoors, buried	mummification/skeletonization	not indicated	Bianchini e Manfrini 1924
Ascomycota	*Geotrichum candidum* Link	UK	not indicated	not indicated	not indicated	Hawksworth & Wiltshire 2015
Ascomycota	*Geotrichum* sp.	Brazil	indoors	fresh	not indicated	Sidrim et al. 2010
Ascomycota	*Geotrichum* sp.	UK	outdoors, water	not indicated	at least 5 weeks in water	Hawksworth & Wiltshire 2011
Ascomycota	*Gliocladium* sp.	Japan	outdoors	mummification/skeletonization	>6 months	Ishii et al. 2006
Ascomycota	*Gloeotinia M.* Wilson, Noble & E.G. Gray	Italy	outdoors, buried	mummification/skeletonization	not indicated	Bianchini e Manfrini 1924
Ascomycota	*Gongronella butleri* (Lendn.) Peyronel & Dal Vesco	Italy	outdoors, buried	mummification/skeletonization	not indicated	Ciferri R 1943
Ascomycota	*Graphiopsis chlorocephala* (Fresen.) Trail	Italy	outdoors, buried	mummification/skeletonization	not indicated	Bianchini e Manfrini 1924
Ascomycota	*Gymnascella aurantiaca* Peck	Italy	outdoors, buried	mummification/skeletonization	not indicated	Bianchini e Manfrini 1924
Ascomycota	*Gymnascella aurantiaca* Peck	Italy	outdoors, buried	mummification/skeletonization	not indicated	Bianchini e Manfrini 1924
Ascomycota	*Gymnoascoideus* sp.	Italy	indoors	mummification/skeletonization	more than 100 years	Piñar et al. 2013
Ascomycota	*Hamigera avellanea* (Thom & Turesson) Stolk & Samson	Italy	outdoors, buried	mummification/skeletonization	not indicated	Ciferri R 1943
Ascomycota	*Hanseniaspora uvarum* (Niehaus) Shehata, Mrak & Phaff ex M.T. Sm.	Germany	outdoors, buried	not indicated	2 months	Dósa 1955
Ascomycota	*Hanseniaspora uvarum* (Niehaus) Shehata, Mrak & Phaff ex M.T. Sm.	Germany	outdoors, buried	not indicated	48 days	Dósa 1955
Ascomycota	*Hanseniaspora uvarum* (Niehaus) Shehata, Mrak & Phaff ex M.T. Sm.	Germany	outdoors, buried	not indicated	10 days	Dósa 1955
Ascomycota	*Hanseniaspora uvarum* (Niehaus) Shehata, Mrak & Phaff ex M.T. Sm.	Germany	outdoors, buried	not indicated	1 month	Dósa 1955
Ascomycota	*Hormodendrum* sp.	Holland	indoors	not indicated	several weeks	van de Voorde & van Dijck 1982
Ascomycota	*Hyalopus geophilus* Sacc. Et Peyr.	Italy	outdoors, buried	fresh/decay	not indicated	Ciferri R 1943
Ascomycota	*Hypomyces aurantius* (Pers.) Tul.	Germany	outdoors, buried	not indicated	2 years	Dósa 1955
Ascomycota	*Hypomyces aurantius* (Pers.) Tul.	Germany	outdoors, buried	not indicated	2 years	Dósa 1955
Ascomycota	*Hypomyces aurantius* (Pers.) Tul.	Germany	outdoors, buried	not indicated	2 years	Dósa 1955
Ascomycota	*Kylindria zignoellae* (Höhn.) DiCosmo, S.M. Berch & W.B. Kendr.	Italy	outdoors, buried	mummification/skeletonization	not indicated	Ciferri R 1943
Ascomycota	*Kylindria zignoellae* (Höhn.) DiCosmo, S.M. Berch & W.B. Kendr.	Italy	outdoors, buried	mummification/skeletonization	not indicated	Ciferri R 1943
Ascomycota	*Lecanicillium muscarium* (Petch) Zare & W. Gams	Poland	outdoors	advanced decay	-	Spychała et al. 2026 b
Ascomycota	*Lodderomyces parapsilosis* (Ashford) Q.M. Wang, Boekhout, Yurkov, G. Péter & F.Y. Bai	Brazil	indoors	fresh	not indicated	Sidrim et al. 2010
Ascomycota	*Lodderomyces parapsilosis* (Ashford) Q.M. Wang, Boekhout, Yurkov, G. Péter & F.Y. Bai	Brazil	indoors	fresh	not indicated	Sidrim et al. 2010
Ascomycota	*Lodderomyces parapsilosis* (Ashford) Q.M. Wang, Boekhout, Yurkov, G. Péter & F.Y. Bai	Brazil	indoors	fresh	not indicated	Sidrim et al. 2010
Ascomycota	*Lodderomyces parapsilosis* (Ashford) Q.M. Wang, Boekhout, Yurkov, G. Péter & F.Y. Bai	Brazil	indoors	fresh	not indicated	Sidrim et al. 2010
Ascomycota	*Melanopsamma ossicola* Tassi	Italy	outdoors, buried	mummification/skeletonization	not indicated	Ciferri R 1943
Ascomycota	*Meyerozyma guilliermondii* (Wick.) Kurtzman & M. Suzuki	Brazil	-	advanced decay	not indicated	Sidrim et al. 2010
Ascomycota	*Monodictys antiqua* (Corda) S. Hughes	Italy	outdoors, buried	mummification/skeletonization	not indicated	Ciferri R 1943
Ascomycota	*Monodictys castaneae* (Wallr.) S. Hughes	Italy	outdoors, buried	advanced decay	not indicated	Bianchini e Manfrini 1924
Ascomycota	*Monodictys castaneae* (Wallr.) S. Hughes	Italy	-	advanced decay	not indicated	Bianchini, G 1923
Ascomycota	*Monosporium acuminatum* “Bon. var. terrestre Sacc.”	Italy	-	mummification/skeletonization	not indicated	Bianchini, G 1923
Ascomycota	*Monosporium acuminatum* var. terrestre Bon. Sacc.	Italy	outdoors, buried	mummification/skeletonization	not indicated	Bianchini e Manfrini 1924
Ascomycota	*Mycogone perniciosa* Magnus ex Delacr.	Germany	outdoors, buried	not indicated	4 years	Dósa 1955
Ascomycota	*Mycogone perniciosa* Magnus ex Delacr.	Germany	outdoors, buried	not indicated	3 years	Dósa 1955
Ascomycota	*Nakaseomyces glabratus* (H.W. Anderson) Sugita & M. Takash.	Germany	indoors	decay/mummification/skeletonization	1–2 weeks	Schwarz et al. 2015
Ascomycota	*Neonectria lugdunensis* (Sacc. & Therry) L. Lombard & Crous	Germany	outdoors, water	advanced decay	>3 days	Piepenbring, M 2025
Ascomycota	*Neonectria lugdunensis* (Sacc. & Therry) L. Lombard & Crous	Poland	outdoors	advanced decay	-	Spychała et al. 2026 b
Ascomycota	*Neurospora sitophila* Shear & B.O. Dodge	Thailand	indoors	not indicated	not indicated (long)	Lakchayapakorn et al. 2008
Ascomycota	*Oidiodendron truncatum* G.L. Barron	Poland	outdoors	advanced decay	-	Spychała et al. 2026 b
Ascomycota	*Oidiodendron truncatum* G.L. Barron	Poland	outdoors	advanced decay	-	Spychała et al. 2026 b
Ascomycota	*Oidiodendron truncatum* G.L. Barron	Poland	outdoors	advanced decay	-	Spychała et al. 2026 b
Ascomycota	*Oidiodendron truncatum* G.L. Barron	Poland	outdoors	advanced decay	-	Spychała et al. 2026 b
Ascomycota	*Oidiodendron truncatum* G.L. Barron	Poland	outdoors	advanced decay	-	Spychała et al. 2026 b
Ascomycota	*Oidiodendron truncatum* G.L. Barron	Poland	outdoors	advanced decay	-	Spychała et al. 2026 b
Ascomycota	*Oidiodendron truncatum* G.L. Barron	Poland	outdoors	advanced decay	-	Spychała et al. 2026 b
Ascomycota	*Oidium inaequale* Rivolta	Italy	outdoors, buried	fresh/decay	not indicated	Ciferri R 1943
Ascomycota	*Oidium inaequale* Rivolta	Italy	outdoors, buried	advanced decay	not indicated	Ciferri R 1943
Ascomycota	*Oidium inaequale* Rivolta	Italy	-	fresh/decay	not indicated	Bianchini, G 1923
Ascomycota	*Oidium penicillioides* Rivolta	Italy	outdoors, buried	fresh/decay	not indicated	Ciferri R 1943
Ascomycota	*Oidium penicillioides* Rivolta	Italy	outdoors, buried	fresh/decay	not indicated	Ciferri R 1943
Ascomycota	*Oidium penicillioides* Rivolta	Italy	outdoors, buried	fresh/decay	not indicated	Ciferri R 1943
Ascomycota	*Oidium penicillioides* Rivolta	Italy	outdoors, buried	fresh/decay	not indicated	Ciferri R 1943
Ascomycota	*Oidium penicillioides* Rivolta	Italy	outdoors, buried	fresh/decay	not indicated	Ciferri R 1943
Ascomycota	*Oidium penicillioides* Rivolta	Italy	outdoors, buried	fresh/decay	not indicated	Ciferri R 1943
Ascomycota	*Oidium penicillioides* Rivolta	Italy	outdoors, buried	fresh/decay	not indicated	Ciferri R 1943
Ascomycota	*Oidium penicillioides* Rivolta	Italy	-	fresh/decay	not indicated	Bianchini, G 1923
Ascomycota	*Onygena apus* Berk. Et Broome	Italy	outdoors, buried	mummification/skeletonization	not indicated	Bianchini e Manfrini 1924
Ascomycota	*Oospora sulfurea* Sacc. & Roum.	Italy	outdoors, buried	advanced decay	not indicated	Ciferri R 1943
Ascomycota	*Oospora sulfurea* Sacc. & Roum.	Italy	outdoors, buried	advanced decay	not indicated	Ciferri R 1943
Ascomycota	*Oospora sulphurea* (Preuss) Sacc. & Voglino,	Italy	-	advanced decay	not indicated	Bianchini, G 1923
Ascomycota	*Paecilomyces niveus* Stolk & Samson	UK	not indicated	not indicated	not indicated	Hawksworth & Wiltshire 2015
Ascomycota	*Paracremonium apiculatum* Z.F. Zhang & L. Cai	Italy	outdoors, buried	mummification/skeletonization	not indicated	Bianchini e Manfrini 1924
Ascomycota	*Paracremonium apiculatum* Z.F. Zhang & L. Cai	Italy	-	mummification/skeletonization	not indicated	Bianchini, G 1923
Ascomycota	*Penicillium albicans* Bain.	Italy	outdoors, buried	fresh/decay	not indicated	Bianchini e Manfrini 1924
Ascomycota	*Penicillium albicans* Bainier	Italy	outdoors, buried	mummification/skeletonization	not indicated	Bianchini e Manfrini 1924
Ascomycota	*Penicillium allii* Vincent & Pitt	Poland	outdoors	advanced decay	not indicated	Spychała, K 2025
Ascomycota	*Penicillium atramentosum* Thom	Poland	outdoors	fresh stage	-	Spychała et al. 2026 b
Ascomycota	*Penicillium atramentosum* Thom	Poland	outdoors	fresh stage	-	Spychała et al. 2026 b
Ascomycota	*Penicillium atramentosum* Thom	Poland	outdoors	advanced postmortem changes	-	Spychała et al. 2026 b
Ascomycota	*Penicillium atramentosum* Thom	Poland	outdoors	advanced postmortem changes	-	Spychała et al. 2026 b
Ascomycota	*Penicillium atramentosum* Thom	Poland	outdoors	advanced postmortem changes	-	Spychała et al. 2026 b
Ascomycota	*Penicillium atramentosum* Thom	Poland	outdoors	advanced postmortem changes	-	Spychała et al. 2026 b
Ascomycota	*Penicillium atramentosum* Thom	Poland	outdoors	advanced postmortem changes	-	Spychała et al. 2026 b
Ascomycota	*Penicillium atramentosum* Thom	Poland	outdoors	advanced postmortem changes	-	Spychała et al. 2026 b
Ascomycota	*Penicillium atramentosum* Thom	Poland	outdoors	advanced postmortem changes	-	Spychała et al. 2026 b
Ascomycota	*Penicillium atramentosum* Thom	Poland	outdoors	advanced postmortem changes	-	Spychała et al. 2026 b
Ascomycota	*Penicillium atramentosum* Thom	Poland	outdoors	advanced postmortem changes	-	Spychała et al. 2026 b
Ascomycota	*Penicillium atramentosum* Thom	Poland	outdoors	advanced postmortem changes	-	Spychała et al. 2026 b
Ascomycota	*Penicillium atramentosum* Thom	Poland	outdoors	advanced postmortem changes	-	Spychała et al. 2026 b
Ascomycota	*Penicillium atramentosum* Thom	Poland	outdoors	decay with signs of mummification	-	Spychała et al. 2026 b
Ascomycota	*Penicillium bialowiezense* K. Zaleski, Bull. Int. Acad. Polon. Sci., Cl. Sci. Math., Sér. B.	Poland	outdoors	fresh stage	-	Spychała et al. 2026 b
Ascomycota	*Penicillium brevicompactum* Dierckx	Germany	indoors	fresh	17 days	Piepenbring, M 2025
Ascomycota	*Penicillium brevicompactum* Dierckx	Germany	-	advanced decay	not indicated	Schwarz et al. 2015
Ascomycota	*Penicillium brevicompactum* Dierckx	Germany	indoors	advanced decay	2 months	Schwarz et al. 2015
Ascomycota	*Penicillium brevicompactum* Dierckx	Italy	indoors	mummification/skeletonization	more than 100 years	Piñar et al. 2013
Ascomycota	*Penicillium brevicompactum* Dierckx	Italy	indoors	advanced decay	-	Iannaccone et al. 2026 current
Ascomycota	*Penicillium brevicompactum* Dierckx	UK	not indicated	not indicated	not indicated	Hawksworth & Wiltshire 2015
Ascomycota	*Penicillium camemberti* Thom	Germany	outdoors, buried	not indicated	3 days	Dósa 1955
Ascomycota	*Penicillium camemberti* Thom	Germany	outdoors, buried	not indicated	2 months	Dósa 1955
Ascomycota	*Penicillium camemberti* Thom	Germany	outdoors, buried	not indicated	48 days	Dósa 1955
Ascomycota	*Penicillium camemberti* Thom	Germany	outdoors, buried	not indicated	10 days	Dósa 1955
Ascomycota	*Penicillium carneum* (Frisvad) Frisvad	Germany	outdoors, buried	advanced decay	117 days	Piepenbring, M 2025
Ascomycota	*Penicillium chrysogenum* Thom	Germany	indoors	fresh	not indicated	Piepenbring, M 2025
Ascomycota	*Penicillium chrysogenum* Thom	Germany	indoors	decay/mummification/skeletonization	6 days	Piepenbring, M 2025
Ascomycota	*Penicillium chrysogenum* Thom	Germany	indoors	decay/mummification/skeletonization	66 days	Piepenbring, M 2025
Ascomycota	*Penicillium chrysogenum* Thom	Germany	indoors	advanced decay	not indicated	Schwarz et al. 2015
Ascomycota	*Penicillium chrysogenum* Thom	Germany	indoors	advanced decay	not indicated	Schwarz et al. 2015
Ascomycota	*Penicillium chrysogenum* Thom	Holland	indoors	not indicated	several weeks	van de Voorde & van Dijck 1982
Ascomycota	*Penicillium chrysogenum* Thom	Italy	outdoors	decay/mummification/skeletonization	6 days	Bellini et al. 2015
Ascomycota	*Penicillium chrysogenum* Thom	Italy	indoors	mummification/skeletonization	more than 100 years	Piñar et al. 2013
Ascomycota	*Penicillium chrysogenum* Thom	Italy	indoors	advanced decay	-	Iannaccone et al. 2026 current
Ascomycota	*Penicillium chrysogenum* Thom	Poland	outdoors	advanced decay	not indicated	Spychała, K 2025
Ascomycota	*Penicillium chrysogenum* Thom	Poland	outdoors	advanced decay	-	Spychała et al. 2026 b
Ascomycota	*Penicillium chrysogenum* Thom	Poland	outdoors	fresh stage	-	Spychała et al. 2026 b
Ascomycota	*Penicillium chrysogenum* Thom	Poland	outdoors	advanced decay	-	Spychała et al. 2026 b
Ascomycota	*Penicillium chrysogenum* Thom	Poland	outdoors	advanced decay	-	Spychała et al. 2026 b
Ascomycota	*Penicillium chrysogenum* Thom	Poland	outdoors	advanced postmortem changes	-	Spychała et al. 2026 b
Ascomycota	*Penicillium chrysogenum* Thom	Poland	outdoors	advanced postmortem changes	-	Spychała et al. 2026 b
Ascomycota	*Penicillium chrysogenum* Thom	Poland	outdoors	advanced postmortem changes	-	Spychała et al. 2026 b
Ascomycota	*Penicillium chrysogenum* Thom	Poland	outdoors	advanced postmortem changes	-	Spychała et al. 2026 b
Ascomycota	*Penicillium chrysogenum* Thom	Poland	outdoors	advanced postmortem changes	-	Spychała et al. 2026 b
Ascomycota	*Penicillium chrysogenum* Thom	Poland	outdoors	advanced postmortem changes	-	Spychała et al. 2026 b
Ascomycota	*Penicillium chrysogenum* Thom	Poland	outdoors	advanced postmortem changes	-	Spychała et al. 2026 b
Ascomycota	*Penicillium chrysogenum* Thom	Poland	outdoors	advanced postmortem changes	-	Spychała et al. 2026 b
Ascomycota	*Penicillium chrysogenum* Thom	Poland	outdoors	advanced postmortem changes	-	Spychała et al. 2026 b
Ascomycota	*Penicillium chrysogenum* Thom	Poland	outdoors	decay with signs of mummification	-	Spychała et al. 2026 b
Ascomycota	*Penicillium chrysogenum* Thom	Poland	outdoors	decay with signs of mummification	-	Spychała et al. 2026 b
Ascomycota	*Penicillium chrysogenum* Thom	Poland	outdoors	decay with signs of mummification	-	Spychała et al. 2026 b
Ascomycota	*Penicillium chrysogenum* Thom	Poland	outdoors	decay with signs of mummification	-	Spychała et al. 2026 b
Ascomycota	*Penicillium chrysogenum* Thom	Poland	outdoors	decay with signs of mummification	-	Spychała et al. 2026 b
Ascomycota	*Penicillium citrinum* Thom	UK	not indicated	not indicated	not indicated	Hawksworth & Wiltshire 2015
Ascomycota	*Penicillium clavigerum*	Poland	outdoors, close to water	decay/mummification/skeletonization	approximately 4 months	Spychała et al. 2026 a
Ascomycota	*Penicillium commune* Thom	Japan	outdoors	advanced decay	4 months	Kato et al. 2023
Ascomycota	*Penicillium commune* Thom	Poland	outdoors	advanced decay	not indicated	Spychała, K 2025
Ascomycota	*Penicillium commune* Thom	Poland	outdoors	fresh stage	-	Spychała et al. 2026 b
Ascomycota	*Penicillium commune* Thom	Poland	outdoors	fresh stage	-	Spychała et al. 2026 b
Ascomycota	*Penicillium commune* Thom	Poland	outdoors	fresh stage	-	Spychała et al. 2026 b
Ascomycota	*Penicillium commune* Thom	Poland	outdoors	fresh stage	-	Spychała et al. 2026 b
Ascomycota	*Penicillium commune* Thom	Poland	outdoors	fresh stage	-	Spychała et al. 2026 b
Ascomycota	*Penicillium commune* Thom	Poland	outdoors	advanced postmortem changes	-	Spychała et al. 2026 b
Ascomycota	*Penicillium commune* Thom	Poland	outdoors	advanced postmortem changes	-	Spychała et al. 2026 b
Ascomycota	*Penicillium commune* Thom	Poland	outdoors	advanced postmortem changes	-	Spychała et al. 2026 b
Ascomycota	*Penicillium commune* Thom	Poland	outdoors	advanced postmortem changes	-	Spychała et al. 2026 b
Ascomycota	*Penicillium commune* Thom	Poland	outdoors	advanced postmortem changes	-	Spychała et al. 2026 b
Ascomycota	*Penicillium commune* Thom	Poland	outdoors	advanced postmortem changes	-	Spychała et al. 2026 b
Ascomycota	*Penicillium commune* Thom	Poland	outdoors	advanced postmortem changes	-	Spychała et al. 2026 b
Ascomycota	*Penicillium commune* Thom	Poland	outdoors	advanced postmortem changes	-	Spychała et al. 2026 b
Ascomycota	*Penicillium commune* Thom	Poland	outdoors	advanced postmortem changes	-	Spychała et al. 2026 b
Ascomycota	*Penicillium commune* Thom	Poland	outdoors	advanced postmortem changes	-	Spychała et al. 2026 b
Ascomycota	*Penicillium commune* Thom	Poland	outdoors	decay with signs of mummification	-	Spychała et al. 2026 b
Ascomycota	*Penicillium commune* Thom	Poland	outdoors	decay with signs of mummification	-	Spychała et al. 2026 b
Ascomycota	*Penicillium commune* Thom	Poland	outdoors	decay with signs of mummification	-	Spychała et al. 2026 b
Ascomycota	*Penicillium corylophilum* Dierckx	Poland	outdoors	fresh stage	-	Spychała et al. 2026 b
Ascomycota	*Penicillium crustaceum* (L.) Fr.	Germany	outdoors, buried	not indicated	2 years	Dósa 1955
Ascomycota	*Penicillium crustaceum* (L.) Fr.	Germany	outdoors, buried	not indicated	3 years	Dósa 1955
Ascomycota	*Penicillium crustaceum* (L.) Fr.	Germany	outdoors, buried	not indicated	2 months	Dósa 1955
Ascomycota	*Penicillium crustaceum* (L.) Fr.	Germany	outdoors, buried	not indicated	2 years	Dósa 1955
Ascomycota	*Penicillium crustaceum* (L.) Fr.	Germany	outdoors, buried	not indicated	3 months	Dósa 1955
Ascomycota	*Penicillium crustaceum* (L.) Fr.	Germany	outdoors, buried	not indicated	2 years	Dósa 1955
Ascomycota	*Penicillium crustaceum* (L.) Fr.	Germany	outdoors, buried	not indicated	2 years	Dósa 1955
Ascomycota	*Penicillium crustaceum* (L.) Fr.	Germany	outdoors, buried	not indicated	3 days	Dósa 1955
Ascomycota	*Penicillium crustaceum* (L.) Fr.	Germany	outdoors, buried	not indicated	3 months	Dósa 1955
Ascomycota	*Penicillium crustaceum* (L.) Fr.	Germany	outdoors, buried	not indicated	1 month	Dósa 1955
Ascomycota	*Penicillium crustaceum* (L.) Fr.	Italy	outdoors, buried	fresh/decay	not indicated	Ciferri R 1943
Ascomycota	*Penicillium crustosum* Thom	Germany	outdoors	fresh	7 days	Piepenbring, M 2025
Ascomycota	*Penicillium crustosum* Thom	Germany	indoors	mummification/skeletonization	>75 days	Piepenbring, M 2025
Ascomycota	*Penicillium crustosum* Thom	Germany	indoors	advanced decay	>13 days	Piepenbring, M 2025
Ascomycota	*Penicillium crustosum* Thom	Germany	indoors	advanced decay	17 days	Piepenbring, M 2025
Ascomycota	*Penicillium crustosum* Thom	Germany	indoors	advanced decay	>8 days	Piepenbring, M 2025
Ascomycota	*Penicillium crustosum* Thom	Germany	indoors	advanced decay	>30 days	Piepenbring, M 2025
Ascomycota	*Penicillium crustosum* Thom	Germany	indoors	advanced decay	>17 days	Piepenbring, M 2025
Ascomycota	*Penicillium crustosum* Thom	Germany	outdoors, buried	advanced decay	121 days	Piepenbring, M 2025
Ascomycota	*Penicillium crustosum* Thom	Germany	indoors	decay/mummification/skeletonization	not indicated	Schwarz et al. 2015
Ascomycota	*Penicillium crustosum* Thom	Germany	indoors	advanced decay	4–6 days	Schwarz et al. 2015
Ascomycota	*Penicillium crustosum* Thom	Poland	outdoors	advanced decay	not indicated	Spychała, K 2025
Ascomycota	*Penicillium crustosum* Thom	Poland	outdoors	fresh stage	-	Spychała et al. 2026 b
Ascomycota	*Penicillium crustosum* Thom	Poland	outdoors	advanced decay	-	Spychała et al. 2026 b
Ascomycota	*Penicillium crustosum* Thom	Poland	outdoors	fresh stage	-	Spychała et al. 2026 b
Ascomycota	*Penicillium crustosum* Thom	Poland	outdoors	fresh stage	-	Spychała et al. 2026 b
Ascomycota	*Penicillium crustosum* Thom	Poland	outdoors	fresh stage	-	Spychała et al. 2026 b
Ascomycota	*Penicillium crustosum* Thom	Poland	outdoors	fresh stage	-	Spychała et al. 2026 b
Ascomycota	*Penicillium crustosum* Thom	Poland	outdoors	fresh stage	-	Spychała et al. 2026 b
Ascomycota	*Penicillium crustosum* Thom	Poland	outdoors	fresh stage	-	Spychała et al. 2026 b
Ascomycota	*Penicillium crustosum* Thom	Poland	outdoors	fresh stage	-	Spychała et al. 2026 b
Ascomycota	*Penicillium crustosum* Thom	Poland	outdoors	fresh stage	-	Spychała et al. 2026 b
Ascomycota	*Penicillium crustosum* Thom	Poland	outdoors	fresh stage	-	Spychała et al. 2026 b
Ascomycota	*Penicillium crustosum* Thom	Poland	outdoors	advanced postmortem changes	-	Spychała et al. 2026 b
Ascomycota	*Penicillium crustosum* Thom	Poland	outdoors	advanced postmortem changes	-	Spychała et al. 2026 b
Ascomycota	*Penicillium crustosum* Thom	Poland	outdoors	advanced postmortem changes	-	Spychała et al. 2026 b
Ascomycota	*Penicillium crustosum* Thom	Poland	outdoors	advanced postmortem changes	-	Spychała et al. 2026 b
Ascomycota	*Penicillium crustosum* Thom	Poland	outdoors	advanced postmortem changes	-	Spychała et al. 2026 b
Ascomycota	*Penicillium crustosum* Thom	Poland	outdoors	advanced postmortem changes	-	Spychała et al. 2026 b
Ascomycota	*Penicillium crustosum* Thom	Poland	outdoors	advanced postmortem changes	-	Spychała et al. 2026 b
Ascomycota	*Penicillium crustosum* Thom	Poland	outdoors	advanced postmortem changes	-	Spychała et al. 2026 b
Ascomycota	*Penicillium crustosum* Thom	Poland	outdoors	advanced postmortem changes	-	Spychała et al. 2026 b
Ascomycota	*Penicillium crustosum* Thom	Poland	outdoors	advanced postmortem changes	-	Spychała et al. 2026 b
Ascomycota	*Penicillium crustosum* Thom	Poland	outdoors	advanced postmortem changes	-	Spychała et al. 2026 b
Ascomycota	*Penicillium crustosum* Thom	Poland	outdoors	decay with signs of mummification	-	Spychała et al. 2026 b
Ascomycota	*Penicillium crustosum* Thom	Poland	outdoors	decay with signs of mummification	-	Spychała et al. 2026 b
Ascomycota	*Penicillium digitatum* (Pers.) Sacc.	Italy	outdoors, buried	fresh/decay	not indicated	Ciferri R 1943
Ascomycota	*Penicillium digitatum* (Pers.) Sacc.	Italy	-	fresh/decay	not indicated	Bianchini, G 1923
Ascomycota	*Penicillium discolor* Frisvad & Samson	Poland	outdoors	advanced decay	not indicated	Spychała, K 2025
Ascomycota	*Penicillium expansum* Link	Germany	outdoors, buried	not indicated	2 weeks	Dósa 1955
Ascomycota	*Penicillium expansum* Link	Germany	outdoors, buried	not indicated	48 days	Dósa 1955
Ascomycota	*Penicillium expansum* Link	Germany	outdoors, buried	not indicated	54 days	Dósa 1955
Ascomycota	*Penicillium expansum* Link	Germany	outdoors, buried	not indicated	10 days	Dósa 1955
Ascomycota	*Penicillium expansum* Link	Germany	indoors	decay/mummification/skeletonization	not indicated	Schwarz et al. 2015
Ascomycota	*Penicillium expansum* Link	Germany	indoors	advanced decay	2 months	Schwarz et al. 2015
Ascomycota	*Penicillium expansum* Link	Italy	indoors	not indicated	7 days	Di Piazza et al. 2018
Ascomycota	*Penicillium expansum* Link	Italy	indoors	mummification/skeletonization	more than 100 years	Piñar et al. 2013
Ascomycota	*Penicillium expansum* Link	Poland	outdoors	advanced decay	not indicated	Spychała, K 2025
Ascomycota	*Penicillium expansum* Link	Poland	outdoors	decay with signs of mummification	-	Spychała et al. 2026 b
Ascomycota	*Penicillium fuscoglaucum* Biourge	Poland	outdoors	advanced decay	not indicated	Spychała, K 2025
Ascomycota	*Penicillium glabrum* (Wehmer) Westling	Germany	indoors	fresh	11 days	Piepenbring, M 2025
Ascomycota	*Penicillium glabrum* (Wehmer) Westling	Italy	outdoors, buried	mummification/skeletonization	not indicated	Bianchini e Manfrini 1924
Ascomycota	*Penicillium glabrum* (Wehmer) Westling	Italy	outdoors, buried	mummification/skeletonization	not indicated	Bianchini e Manfrini 1924
Ascomycota	*Penicillium griseofulvum*	Poland	outdoors, close to water	decay/mummification/skeletonization	approximately 4 months	Spychała et al. 2026 a
Ascomycota	*Penicillium griseofulvum* Dierckx	Germany	indoors	advanced decay	8 days	Piepenbring, M 2025
Ascomycota	*Penicillium griseofulvum* Dierckx	Germany	outdoors, close to water	advanced decay	>11 days	Piepenbring, M 2025
Ascomycota	*Penicillium griseofulvum* Dierckx	Poland	outdoors	advanced postmortem changes	-	Spychała et al. 2026 b
Ascomycota	*Penicillium griseofulvum* Dierckx	UK	not indicated	not indicated	not indicated	Hawksworth & Wiltshire 2015
Ascomycota	*Penicillium hordei* Stolk	Poland	outdoors	advanced postmortem changes	-	Spychała et al. 2026 b
Ascomycota	*Penicillium italicum* Wehmer	Germany	outdoors, buried	decay/mummification/skeletonization	117 days	Piepenbring, M 2025
Ascomycota	*Penicillium magnielliptisporum* Visagie, Seifert & Samson	Poland	outdoors	advanced decay	not indicated	Spychała, K 2025
Ascomycota	*Penicillium magnielliptisporum* Visagie, Seifert & Samson	Poland	outdoors	fresh stage	-	Spychała et al. 2026 b
Ascomycota	*Penicillium magnielliptisporum* Visagie, Seifert & Samson	Poland	outdoors	fresh stage	-	Spychała et al. 2026 b
Ascomycota	*Penicillium magnielliptisporum* Visagie, Seifert & Samson	Poland	outdoors	advanced postmortem changes	-	Spychała et al. 2026 b
Ascomycota	*Penicillium magnielliptisporum* Visagie, Seifert & Samson	Poland	outdoors	advanced postmortem changes	-	Spychała et al. 2026 b
Ascomycota	*Penicillium magnielliptisporum* Visagie, Seifert & Samson	Poland	outdoors	advanced postmortem changes	-	Spychała et al. 2026 b
Ascomycota	*Penicillium magnielliptisporum* Visagie, Seifert & Samson	Poland	outdoors	advanced postmortem changes	-	Spychała et al. 2026 b
Ascomycota	*Penicillium magnielliptisporum* Visagie, Seifert & Samson	Poland	outdoors	advanced postmortem changes	-	Spychała et al. 2026 b
Ascomycota	*Penicillium magnielliptisporum* Visagie, Seifert & Samson	Poland	outdoors	advanced postmortem changes	-	Spychała et al. 2026 b
Ascomycota	*Penicillium magnielliptisporum* Visagie, Seifert & Samson	Poland	outdoors	advanced postmortem changes	-	Spychała et al. 2026 b
Ascomycota	*Penicillium nalgiovense* Laxa	Germany	indoors	advanced decay	>7 days	Piepenbring, M 2025
Ascomycota	*Penicillium olsonii* Bainier & Sartory	Germany	indoors	fresh	17 days	Piepenbring, M 2025
Ascomycota	*Penicillium oxalicum* Currie & Thom	Thailand	indoors	not indicated	not indicated (long)	Sri-Indrasutdhi et al. 2015
Ascomycota	*Penicillium pancosmium* Houbraken, Frisvad & Samson	Italy	outdoors	decay/mummification/skeletonization	6 days	Bellini et al. 2015
Ascomycota	*Penicillium paneum* Frisvad	Poland	outdoors	advanced postmortem changes	-	Spychała et al. 2026 b
Ascomycota	*Penicillium polonicum* K.W. Zaleski	Germany	indoors	advanced decay	not indicated	Schwarz et al. 2015
Ascomycota	*Penicillium polonicum* K.W. Zaleski	Italy	indoors	not indicated	7 days	Di Piazza et al. 2018
Ascomycota	*Penicillium polonicum* K.W. Zaleski	Poland	outdoors	advanced decay	not indicated	Spychała, K 2025
Ascomycota	*Penicillium polonicum* K.W. Zaleski	Poland	outdoors	Fresh stage	-	Spychała et al. 2026 b
Ascomycota	*Penicillium polonicum* K.W. Zaleski	Poland	outdoors	Fresh stage	-	Spychała et al. 2026 b
Ascomycota	*Penicillium polonicum* K.W. Zaleski	Poland	outdoors	Fresh stage	-	Spychała et al. 2026 b
Ascomycota	*Penicillium polonicum* K.W. Zaleski	Poland	outdoors	Fresh stage	-	Spychała et al. 2026 b
Ascomycota	*Penicillium polonicum* K.W. Zaleski	Poland	outdoors	Advanced decay	-	Spychała et al. 2026 b
Ascomycota	*Penicillium polonicum* K.W. Zaleski	Poland	outdoors	Fresh stage	-	Spychała et al. 2026 b
Ascomycota	*Penicillium polonicum* K.W. Zaleski	Poland	outdoors	Advanced postmortem changes	-	Spychała et al. 2026 b
Ascomycota	*Penicillium polonicum* K.W. Zaleski	Poland	outdoors	Advanced postmortem changes	-	Spychała et al. 2026 b
Ascomycota	*Penicillium polonicum* K.W. Zaleski	Poland	outdoors	Advanced postmortem changes	-	Spychała et al. 2026 b
Ascomycota	*Penicillium polonicum* K.W. Zaleski	Poland	outdoors	Decay with signs of mummification	-	Spychała et al. 2026 b
Ascomycota	*Penicillium rubens* Biourge	Germany	indoors	mummification/skeletonization	>75 days	Piepenbring, M 2025
Ascomycota	*Penicillium rubens* Biourge	Poland	outdoors	Advanced postmortem changes	-	Spychała et al. 2026 b
Ascomycota	*Penicillium rubens* Biourge	Poland	outdoors	Advanced postmortem changes	-	Spychała et al. 2026 b
Ascomycota	*Penicillium rubens* Biourge	Poland	outdoors	Advanced postmortem changes	-	Spychała et al. 2026 b
Ascomycota	*Penicillium rubens* Biourge	Poland	outdoors	Advanced postmortem changes	-	Spychała et al. 2026 b
Ascomycota	*Penicillium rubens* Biourge	Poland	outdoors	Advanced postmortem changes	-	Spychała et al. 2026 b
Ascomycota	*Penicillium rubens* Biourge	Poland	outdoors	Decay with signs of mummification	-	Spychała et al. 2026 b
Ascomycota	*Penicillium rubens* Biourge	Poland	outdoors	Decay with signs of mummification	-	Spychała et al. 2026 b
Ascomycota	*Penicillium rubens* Biourge	Poland	outdoors	Decay with signs of mummification	-	Spychała et al. 2026 b
Ascomycota	*Penicillium rubens* Biourge	Poland	outdoors	Decay with signs of mummification	-	Spychała et al. 2026 b
Ascomycota	*Penicillium simplicissimum* (Oudem.) Thom	Poland	outdoors	Advanced decay	-	Spychała et al. 2026 b
Ascomycota	*Penicillium simplicissimum* (Oudem.) Thom	Poland	outdoors	Advanced decay	-	Spychała et al. 2026 b
Ascomycota	*Penicillium simplicissimum* (Oudem.) Thom	Poland	outdoors	Advanced decay	-	Spychała et al. 2026 b
Ascomycota	*Penicillium simplicissimum* (Oudem.) Thom	Poland	outdoors	Advanced decay	-	Spychała et al. 2026 b
Ascomycota	*Penicillium simplicissimum* (Oudem.) Thom	Poland	outdoors	Advanced decay	-	Spychała et al. 2026 b
Ascomycota	*Penicillium solitum* Westling	Germany	outdoors	advanced decay	182 days	Piepenbring, M 2025
Ascomycota	*Penicillium solitum* Westling	Poland	outdoors	advanced decay	not indicated	Spychała, K 2025
Ascomycota	*Penicillium solitum* Westling	Poland	outdoors	Fresh stage	-	Spychała et al. 2026 b
Ascomycota	*Penicillium solitum* Westling	Poland	outdoors	Advanced postmortem changes	-	Spychała et al. 2026 b
Ascomycota	*Penicillium solitum* Westling	Poland	outdoors	Advanced postmortem changes	-	Spychała et al. 2026 b
Ascomycota	*Penicillium solitum* Westling	Poland	outdoors	Advanced postmortem changes	-	Spychała et al. 2026 b
Ascomycota	*Penicillium solitum* Westling	Poland	outdoors	Advanced postmortem changes	-	Spychała et al. 2026 b
Ascomycota	*Penicillium solitum* Westling	Poland	outdoors	Advanced postmortem changes	-	Spychała et al. 2026 b
Ascomycota	*Penicillium* sp.	Brazil	indoors	fresh	not indicated	Sidrim et al. 2010
Ascomycota	*Penicillium* sp.	Brazil	-	advanced decay	not indicated	Sidrim et al. 2010
Ascomycota	*Penicillium* sp.	Brazil	outdoors	mummification/skeletonization	not indicated	Sidrim et al. 2010
Ascomycota	*Penicillium* sp.	Iraq	indoors	not indicated	not indicated (long)	Hammad et al. 2002
Ascomycota	*Penicillium* sp.	Thailand	indoors	not indicated	not indicated (long)	Lakchayapakorn et al. 2008
Ascomycota	*Penicillium speluncae* Visagie & N. Yilmaz	Poland	outdoors	Advanced postmortem changes	-	Spychała et al. 2026 b
Ascomycota	*Penicillium vinaceum* J.C. Gilman & E.V. Abbott	Poland	outdoors	advanced decay	not indicated	Spychała, K 2025
Ascomycota	*Penidiella* sp.	Italy	indoors	mummification/skeletonization	more than 100 years	Piñar et al. 2013
Ascomycota	*Periconia toruloides* Fresen	Italy	outdoors, buried	mummification/skeletonization	not indicated	Ciferri R 1943
Ascomycota	*Pestalotiopsis* sp.	Japan	outdoors	advanced decay	1 month	Kato et al. 2023
Ascomycota	*Phoma herbarum* Westend., Bull. Acad. Roy. Sci. Belgique	Poland	outdoors	Fresh stage	-	Spychała et al. 2026 b
Ascomycota	*Preussia typharum* (Sacc.) Cain	Poland	outdoors	Decay with signs of mummification	-	Spychała et al. 2026 b
Ascomycota	*Probably never* correctly published	Italy	-	mummification/skeletonization	not indicated	Bianchini, G 1923
Ascomycota	*Pseudeurotium bakeri* C. Booth	Germany	outdoors, buried	advanced decay	121 days	Piepenbring, M 2025
Ascomycota	*Pseudeurotium bakeri* C. Booth	Germany	outdoors, buried	advanced decay	121 days	Piepenbring, M 2025
Ascomycota	*Pseudeurotium zonatum* J.F.H. Beyma	Poland	outdoors	Advanced decay	-	Spychała et al. 2026 b
Ascomycota	*Pseudeurotium zonatum* J.F.H. Beyma	Poland	outdoors	Advanced decay	-	Spychała et al. 2026 b
Ascomycota	*Pseudeurotium zonatum* J.F.H. Beyma	Poland	outdoors	Advanced decay	-	Spychała et al. 2026 b
Ascomycota	*Pseudogymnoascus pannorum* (Link) Minnis & D.L. Lindner	Germany	-	advanced decay	not indicated	Schwarz et al. 2015
Ascomycota	*Pseudogymnoascus pannorum* (Link) Minnis & D.L. Lindner	Poland	outdoors	advanced decay	not indicated	Spychała, K 2025
Ascomycota	*Pseudogymnoascus pannorum* (Link) Minnis & D.L. Lindner	Poland	outdoors	Fresh stage	-	Spychała et al. 2026 b
Ascomycota	*Pseudogymnoascus pannorum* (Link) Minnis & D.L. Lindner	Poland	outdoors	Fresh stage	-	Spychała et al. 2026 b
Ascomycota	*Pseudogymnoascus pannorum* (Link) Minnis & D.L. Lindner	Poland	outdoors	Advanced decay	-	Spychała et al. 2026 b
Ascomycota	*Pseudogymnoascus pannorum* (Link) Minnis & D.L. Lindner	Poland	outdoors	Advanced decay	-	Spychała et al. 2026 b
Ascomycota	*Pseudogymnoascus pannorum* (Link) Minnis & D.L. Lindner	Poland	outdoors	Advanced decay	-	Spychała et al. 2026 b
Ascomycota	*Pseudogymnoascus pannorum* (Link) Minnis & D.L. Lindner	Poland	outdoors	Advanced decay	-	Spychała et al. 2026 b
Ascomycota	*Pseudogymnoascus pannorum* (Link) Minnis & D.L. Lindner	Poland	outdoors	Advanced decay	-	Spychała et al. 2026 b
Ascomycota	*Pseudogymnoascus pannorum* (Link) Minnis & D.L. Lindner	Poland	outdoors	Advanced decay	-	Spychała et al. 2026 b
Ascomycota	*Pseudogymnoascus pannorum* (Link) Minnis & D.L. Lindner	Poland	outdoors	Advanced decay	-	Spychała et al. 2026 b
Ascomycota	*Pseudogymnoascus pannorum* (Link) Minnis & D.L. Lindner	Poland	outdoors	Advanced decay	-	Spychała et al. 2026 b
Ascomycota	*Pseudogymnoascus pannorum* (Link) Minnis & D.L. Lindner	Poland	outdoors	Advanced decay	-	Spychała et al. 2026 b
Ascomycota	*Pseudogymnoascus pannorum* (Link) Minnis & D.L. Lindner	Poland	outdoors	Advanced decay	-	Spychała et al. 2026 b
Ascomycota	*Pseudogymnoascus pannorum* (Link) Minnis & D.L. Lindner	Poland	outdoors	Advanced decay	-	Spychała et al. 2026 b
Ascomycota	*Pseudogymnoascus pannorum* (Link) Minnis & D.L. Lindner	Poland	outdoors	Advanced decay	-	Spychała et al. 2026 b
Ascomycota	*Pseudogymnoascus pannorum* (Link) Minnis & D.L. Lindner	Poland	outdoors	Advanced decay	-	Spychała et al. 2026 b
Ascomycota	*Pseudogymnoascus pannorum* (Link) Minnis & D.L. Lindner	Poland	outdoors	Advanced decay	-	Spychała et al. 2026 b
Ascomycota	*Pseudogymnoascus pannorum* (Link) Minnis & D.L. Lindner	Poland	outdoors	Advanced decay	-	Spychała et al. 2026 b
Ascomycota	*Pseudogymnoascus pannorum* (Link) Minnis & D.L. Lindner	Poland	outdoors	Advanced decay	-	Spychała et al. 2026 b
Ascomycota	*Pseudogymnoascus pannorum* (Link) Minnis & D.L. Lindner	Poland	outdoors	Fresh stage	-	Spychała et al. 2026 b
Ascomycota	*Pseudogymnoascus pannorum* (Link) Minnis & D.L. Lindner	Poland	outdoors	Advanced postmortem changes	-	Spychała et al. 2026 b
Ascomycota	*Pseudogymnoascus pannorum* (Link) Minnis & D.L. Lindner	UK	not indicated	not indicated	not indicated	Hawksworth & Wiltshire 2015
Ascomycota	*Pseudogymnoascus roseus* Raillo	Germany	outdoors, water	advanced decay	>7 days	Piepenbring, M 2025
Ascomycota	*Pseudogymnoascus roseus* Raillo	Germany	indoors	advanced decay	2 weeks	Schwarz et al. 2015
Ascomycota	*Pseudohumicola subspiralis* (Chivers) X.Wei Wang, P.J. Han, F.Y. Bai & Houbraken	Poland	outdoors	Advanced decay	-	Spychała et al. 2026 b
Ascomycota	*Pseudopithomyces chartarum* (Berk. & M.A. Curtis) Jun F. Li, Ariy. & K.D. Hyde	Poland	outdoors	Decay with signs of mummification	-	Spychała et al. 2026 b
Ascomycota	*Purpureocillium lilacinum* (Thom) Luangsa-ard, Houbraken, Hywel-Jones & Samson	Poland	outdoors	Fresh stage	-	Spychała et al. 2026 b
Ascomycota	*Racodium papyraceum* Pers.	Italy	outdoors, buried	mummification/skeletonization	not indicated	Ciferri R 1943
Ascomycota	*Rhizoctonia* sp.	Switzerland	outdoors, buried	mummification/skeletonization	more than 200 years	Piepenbrink 1986
Ascomycota	*Rhizopus stolonifer* (Ehrenb.) Vuill.	Germany	outdoors, buried	not indicated	2 months	Dósa 1955
Ascomycota	*Rhizopus stolonifer* (Ehrenb.) Vuill.	Germany	outdoors, buried	not indicated	3 months	Dósa 1955
Ascomycota	*Rhizopus stolonifer* (Ehrenb.) Vuill.	Germany	outdoors, buried	not indicated	2 weeks	Dósa 1955
Ascomycota	*Rhizopus stolonifer* (Ehrenb.) Vuill.	Germany	outdoors, buried	not indicated	54 days	Dósa 1955
Ascomycota	*Rhizopus stolonifer* (Ehrenb.) Vuill.	Germany	indoors	decay/mummification/skeletonization	14 days	Piepenbring, M 2025
Ascomycota	*Rhizopus stolonifer* (Ehrenb.) Vuill.	Germany	indoors	decay/mummification/skeletonization	not indicated	Schwarz et al. 2015
Ascomycota	*Saccharomyces cerevisiae* (Desm.) Meyen	Poland	outdoors	Fresh stage	-	Spychała et al. 2026 b
Ascomycota	*Saccharomyces* sp.	Germany	outdoors, buried	not indicated	3 months	Dósa 1955
Ascomycota	*Saccharomyces* sp.	Germany	outdoors, buried	not indicated	54 days	Dósa 1955
Ascomycota	*Saccharomyces* sp.	Germany	outdoors, buried	not indicated	3 months	Dósa 1955
Ascomycota	*Saccharomyces* sp.	Germany	outdoors, buried	not indicated	1 month	Dósa 1955
Ascomycota	*Sagenomella* sp.	Italy	indoors	mummification/skeletonization	more than 100 years	Piñar et al. 2013
Ascomycota	*Sarocladium strictum* (W. Gams) Summerb.	Germany	outdoors, buried	not indicated	4 years	Dósa 1955
Ascomycota	*Sarocladium strictum* (W. Gams) Summerb.	Germany	outdoors, buried	not indicated	10 years	Dósa 1955
Ascomycota	*Sarocladium strictum* (W. Gams) Summerb.	Poland	outdoors	Advanced decay	-	Spychała et al. 2026 b
Ascomycota	*Scedosporium aurantiacum* Gilgado, Cano, Gené & Guarro	Poland	outdoors	Advanced decay	-	Spychała et al. 2026 b
Ascomycota	*Scedosporium aurantiacum* Gilgado, Cano, Gené & Guarro	Poland	outdoors	Advanced decay	-	Spychała et al. 2026 b
Ascomycota	*Scedosporium aurantiacum* Gilgado, Cano, Gené & Guarro	Poland	outdoors	Advanced decay	-	Spychała et al. 2026 b
Ascomycota	*Scedosporium aurantiacum* Gilgado, Cano, Gené & Guarro	Poland	outdoors	Advanced decay	-	Spychała et al. 2026 b
Ascomycota	*Scedosporium boydii* (Shear) Gilgado, Gené, Cano & Guarro	Germany	outdoors, buried	not indicated	11 years	Dósa 1955
Ascomycota	*Scopulariopsis asperula* (Sacc.) S. Hughes,	Poland	outdoors	advanced decay	not indicated	Spychała, K 2025
Ascomycota	*Scopulariopsis brevicaulis* (Sacc.) Bainier	Germany	outdoors, buried	not indicated	2 years	Dósa 1955
Ascomycota	*Scopulariopsis brevicaulis* (Sacc.) Bainier	Germany	outdoors, buried	not indicated	2 years	Dósa 1955
Ascomycota	*Scopulariopsis brevicaulis* (Sacc.) Bainier	Germany	outdoors, buried	not indicated	2 years	Dósa 1955
Ascomycota	*Scopulariopsis brevicaulis* (Sacc.) Bainier	Germany	outdoors, buried	not indicated	2 years	Dósa 1955
Ascomycota	*Scopulariopsis brevicaulis* (Sacc.) Bainier	Germany	outdoors, buried	not indicated	2 years	Dósa 1955
Ascomycota	*Scopulariopsis brevicaulis* (Sacc.) Bainier	Germany	outdoors, buried	not indicated	11 years	Dósa 1955
Ascomycota	*Scopulariopsis brevicaulis* (Sacc.) Bainier	Germany	outdoors, buried	not indicated	3 years	Dósa 1955
Ascomycota	*Scopulariopsis brevicaulis* (Sacc.) Bainier	Germany	indoors	decay/mummification/skeletonization	not indicated	Schwarz et al. 2015
Ascomycota	*Scopulariopsis brevicaulis* (Sacc.) Bainier	Germany	indoors	advanced decay	not indicated	Schwarz et al. 2015
Ascomycota	*Scopulariopsis brevicaulis* (Sacc.) Bainier	Poland	indoors	decay/mummification/skeletonization	approx. 2 years	Szleszkowski et al. 2022
Ascomycota	*Scopulariopsis brevicaulis* (Sacc.) Bainier	Poland	outdoors	advanced decay	not indicated	Spychała, K 2025
Ascomycota	*Scopulariopsis brevicaulis* (Sacc.) Bainier	Poland	outdoors	Advanced decay	-	Spychała et al. 2026 b
Ascomycota	*Scopulariopsis brevicaulis* (Sacc.) Bainier	Poland	outdoors	Decay with signs of mummification	-	Spychała et al. 2026 b
Ascomycota	*Scopulariopsis brevicaulis* (Sacc.) Bainier	Poland	outdoors	Decay with signs of mummification	-	Spychała et al. 2026 b
Ascomycota	*Scopulariopsis brevicaulis* (Sacc.) Bainier	Poland	outdoors	Decay with signs of mummification	-	Spychała et al. 2026 b
Ascomycota	*Scopulariopsis brevicaulis* (Sacc.) Bainier	Poland	outdoors	Decay with signs of mummification	-	Spychała et al. 2026 b
Ascomycota	*Scopulariopsis brevicaulis* (Sacc.) Bainier	Poland	outdoors	Decay with signs of mummification	-	Spychała et al. 2026 b
Ascomycota	*Scopulariopsis brevicaulis* (Sacc.) Bainier	Poland	outdoors	Decay with signs of mummification	-	Spychała et al. 2026 b
Ascomycota	*Scopulariopsis brevicaulis* (Sacc.) Bainier	Poland	outdoors	Decay with signs of mummification	-	Spychała et al. 2026 b
Ascomycota	*Scopulariopsis brevicaulis* (Sacc.) Bainier	Poland	outdoors	Decay with signs of mummification	-	Spychała et al. 2026 b
Ascomycota	*Scopulariopsis candida* Vuill.	Poland	outdoors	Advanced decay	-	Spychała et al. 2026 b
Ascomycota	*Scopulariopsis candida* Vuill.	Poland	outdoors	Advanced decay	-	Spychała et al. 2026 b
Ascomycota	*Scopulariopsis candida* Vuill.	Poland	outdoors	Advanced decay	-	Spychała et al. 2026 b
Ascomycota	*Scopulariopsis candida* Vuill.	Poland	outdoors	Advanced decay	-	Spychała et al. 2026 b
Ascomycota	*Scopulariopsis candida* Vuill.	Poland	outdoors	Advanced postmortem changes	-	Spychała et al. 2026 b
Ascomycota	*Scopulariopsis candida* Vuill.	Poland	outdoors	Advanced postmortem changes	-	Spychała et al. 2026 b
Ascomycota	*Scopulariopsis coprophila* (Cooke & Massee) W. Gams	Italy	outdoors, buried	fresh/decay	not indicated	Ciferri R 1943
Ascomycota	*Scopulariopsis crassa* Z.F. Zhang, F. Liu & L. Cai	Poland	outdoors	advanced decay	not indicated	Spychała, K 2025
Ascomycota	*Scopulariopsis minima* “Sart., Huffsch. Et Mey.”	Italy	outdoors, buried	mummification/skeletonization	not indicated	Ciferri R 1943
Ascomycota	*Scopulariopsis minima* “Sart., Huffsch. Et Mey.”	Italy	outdoors, buried	mummification/skeletonization	not indicated	Ciferri R 1943
Ascomycota	*Scopulariopsis minima* “Sart., Huffsch. Et Mey.”	Italy	outdoors, buried	mummification/skeletonization	not indicated	Ciferri R 1943
Ascomycota	*Scopulariopsis minima* “Sart., Huffsch. Et Mey.”	Italy	outdoors, buried	mummification/skeletonization	not indicated	Ciferri R 1943
Ascomycota	*Scytalidium cuboideum* (Sacc. & Ellis) Sigler & H.J. Kang	Italy	outdoors, buried	mummification/skeletonization	not indicated	Ciferri R 1943
Ascomycota	*Scytalidium cuboideum* (Sacc. & Ellis) Sigler & H.J. Kang	Italy	outdoors, buried	mummification/skeletonization	not indicated	Ciferri R 1943
Ascomycota	*Scytalidium cuboideum* (Sacc. & Ellis) Sigler & H.J. Kang	Italy	outdoors, buried	mummification/skeletonization	not indicated	Ciferri R 1943
Ascomycota	*Scytalidium cuboideum* (Sacc. & Ellis) Sigler & H.J. Kang	Italy	outdoors, buried	mummification/skeletonization	not indicated	Ciferri R 1943
Ascomycota	*Scytalidium cuboideum* (Sacc. & Ellis) Sigler & H.J. Kang	Italy	outdoors, buried	mummification/skeletonization	not indicated	Ciferri R 1943
Ascomycota	*Scytalidium cuboideum* (Sacc. & Ellis) Sigler & H.J. Kang	Italy	outdoors, buried	mummification/skeletonization	not indicated	Ciferri R 1943
Ascomycota	*Sesquicillium candelabrum* (Bonord.) W. Gams	Italy	outdoors, buried	mummification/skeletonization	not indicated	Bianchini e Manfrini 1924
Ascomycota	*Sphaeronaema Fr.* Species not available	Italy	outdoors, buried	mummification/skeletonization	not indicated	Bianchini e Manfrini 1924
Ascomycota	*Stachybotrys chartarum* (Ehrenb.) S. Hughes	Italy	outdoors, buried	mummification/skeletonization	not indicated	Ciferri R 1943
Ascomycota	*Stachybotrys chlorohalonatus* B. Andersen & Thrane	Germany	indoors	advanced decay	14 days	Piepenbring, M 2025
Ascomycota	*Stagonosporopsis* sp.	Germany	indoors	fresh/decay	13 days	Piepenbring, M 2025
Ascomycota	*Stemphylium quercinum* Sacc.	Italy	outdoors, buried	mummification/skeletonization	not indicated	Bianchini e Manfrini 1924
Ascomycota	*Sterigmatocystis nigra* v. Tieghem	Italy	-	-	-	Bianchini e Manfrini 1924
Ascomycota	*Sterigmatocystis ochracea* (cfr. Aspergillus)	Italy	outdoors, buried	mummification/skeletonization	not indicated	Ciferri R 1943
Ascomycota	*Striatibotrys eucylindrosporus* (D.W. Li) L. Lombard & Crous	Switzerland	outdoors, buried	mummification/skeletonization	more than 200 years	Piepenbrink 1986
Ascomycota	*Talaromyces flavus* (Klöcker) Stolk & Samson	Poland	outdoors	Advanced decay	-	Spychała et al. 2026 b
Ascomycota	*Talaromyces flavus* (Klöcker) Stolk & Samson	Poland	outdoors	Advanced decay	-	Spychała et al. 2026 b
Ascomycota	*Talaromyces piceae* (Raper & Fennell) Samson, N. Yilmaz, Houbraken, Spierenb., Seifert, Peterson, Varga & Frisvad	Brazil	indoors	fresh	not indicated	Sidrim et al. 2010
Ascomycota	*Talaromyces piceae* (Raper & Fennell) Samson, N. Yilmaz, Houbraken, Spierenb., Seifert, Peterson, Varga & Frisvad	Brazil	-	advanced decay	not indicated	Sidrim et al. 2010
Ascomycota	*Talaromyces piceae* (Raper & Fennell) Samson, N. Yilmaz, Houbraken, Spierenb., Seifert, Peterson, Varga & Frisvad	Brazil	outdoors	mummification/skeletonization	not indicated	Sidrim et al. 2010
Ascomycota	*Talaromyces piceae* (Raper & Fennell) Samson, N. Yilmaz, Houbraken, Spierenb., Seifert, Peterson, Varga & Frisvad	Brazil	indoors	fresh	not indicated	Sidrim et al. 2010
Ascomycota	*Talaromyces piceae* (Raper & Fennell) Samson, N. Yilmaz, Houbraken, Spierenb., Seifert, Peterson, Varga & Frisvad	Brazil	indoors	fresh	not indicated	Sidrim et al. 2010
Ascomycota	*Talaromyces radicus* (A.D. Hocking & Whitelaw) Samson, Yilmaz, Frisvad & Seifert	Italy	indoors	mummification/skeletonization	more than 100 years	Piñar et al. 2013
Ascomycota	*Talaromyces ruber* (Stoll) N. Yilmaz, Houbraken, Frisvad & Samson	Italy	indoors	not indicated	7 days	Di Piazza et al. 2018
Ascomycota	*Talaromyces rugulosus* (Thom) Samson, N. Yilmaz, Frisvad & Seifert	Brazil	indoors	fresh	not indicated	Sidrim et al. 2010
Ascomycota	*Talaromyces rugulosus* (Thom) Samson, N. Yilmaz, Frisvad & Seifert	Brazil	indoors	fresh	not indicated	Sidrim et al. 2010
Ascomycota	*Talaromyces rugulosus* (Thom) Samson, N. Yilmaz, Frisvad & Seifert	Brazil	indoors	fresh	not indicated	Sidrim et al. 2010
Ascomycota	*Talaromyces rugulosus* (Thom) Samson, N. Yilmaz, Frisvad & Seifert	Brazil	outdoors	mummification/skeletonization	not indicated	Sidrim et al. 2010
Ascomycota	*Talaromyces rugulosus* (Thom) Samson, N. Yilmaz, Frisvad & Seifert	Brazil	indoors	fresh	not indicated	Sidrim et al. 2010
Ascomycota	*Thamnidium elegans* Link	Italy	outdoors, buried	fresh/decay	not indicated	Ciferri R 1943
Ascomycota	*Torula perpusilla* Sacc.	Italy	outdoors, buried	mummification/skeletonization	not indicated	Ciferri R 1943
Ascomycota	*Torulaspora* sp.	Germany	outdoors, buried	not indicated	2 months	Dósa 1955
Ascomycota	*Torulaspora* sp.	Germany	outdoors, buried	not indicated	2 years	Dósa 1955
Ascomycota	*Torulaspora* sp.	Germany	outdoors, buried	not indicated	2 years	Dósa 1955
Ascomycota	*Torulaspora* sp.	Germany	outdoors, buried	not indicated	2 years	Dósa 1955
Ascomycota	*Trichocladium griseum* (Traaen) X.Wei Wang & Houbraken	Poland	outdoors	Advanced decay	-	Spychała et al. 2026 b
Ascomycota	*Trichocladium griseum* (Traaen) X.Wei Wang & Houbraken	Poland	outdoors	Advanced decay	-	Spychała et al. 2026 b
Ascomycota	*Trichocladium griseum* (Traaen) X.Wei Wang & Houbraken	Poland	outdoors	Advanced decay	-	Spychała et al. 2026 b
Ascomycota	*Trichoderma atroviride*	Poland	outdoors, close to water	decay/mummification/skeletonization	approximately 4 months	Spychała et al. 2026 a
Ascomycota	*Trichoderma* sp.	Brazil	indoors	fresh	not indicated	Sidrim et al. 2010
Ascomycota	*Trichoderma* sp.	Brazil	outdoors	mummification/skeletonization	not indicated	Sidrim et al. 2010
Ascomycota	*Trichoderma* sp.	Thailand	indoors	not indicated	not indicated (long)	Lakchayapakorn et al. 2008
Ascomycota	*Trichoderma viride* Pers.	Italy	outdoors, buried	mummification/skeletonization	not indicated	Ciferri R 1943
Ascomycota	*Trichophyton* sp.	Iraq	indoors	not indicated	not indicated (long)	Hammad et al. 2002
Ascomycota	*Trichothecium roseum* Fr.	Germany	outdoors, buried	not indicated	11 years	Dósa 1955
Ascomycota	*Trichothecium roseum* Fr.	Germany	outdoors, buried	not indicated	10 years	Dósa 1955
Ascomycota	*Venustampulla parva* (A.H.S. Br. & G. Sm.) Unter. & Réblová	Poland	outdoors	Advanced postmortem changes	-	Spychała et al. 2026 b
Ascomycota	*Venustampulla parva* (A.H.S. Br. & G. Sm.) Unter. & Réblová	Poland	outdoors	Advanced postmortem changes	-	Spychała et al. 2026 b
Ascomycota	*Yarrowia alimentaria* (Knutsen, V. Robert & M.T. Sm.) Gouliam., R.A. Dimitrov, M.T. Sm. & M. Groenew.	Poland	outdoors	Fresh stage	-	Spychała et al. 2026 b
Ascomycota	*Yarrowia alimentaria* (Knutsen, V. Robert & M.T. Sm.) Gouliam., R.A. Dimitrov, M.T. Sm. & M. Groenew.	Poland	outdoors	Fresh stage	-	Spychała et al. 2026 b
Ascomycota	*Yarrowia alimentaria* (Knutsen, V. Robert & M.T. Sm.) Gouliam., R.A. Dimitrov, M.T. Sm. & M. Groenew.	Poland	outdoors	Fresh stage	-	Spychała et al. 2026 b
Ascomycota	*Yarrowia alimentaria* (Knutsen, V. Robert & M.T. Sm.) Gouliam., R.A. Dimitrov, M.T. Sm. & M. Groenew.	Poland	outdoors	Fresh stage	-	Spychała et al. 2026 b
Ascomycota	*Yarrowia bubula* Ed. Nagy, Dlauchy & G. Péter	Poland	outdoors	Fresh stage	-	Spychała et al. 2026 b
Ascomycota	*Yarrowia deformans*	Poland	outdoors, close to water	decay/mummification/skeletonization	approximately 4 months	Spychała et al. 2026 a
Ascomycota	*Yarrowia deformans*	Poland	outdoors, close to water	decay/mummification/skeletonization	approximately 4 months	Spychała et al. 2026 a
Ascomycota	*Yarrowia deformans*	Poland	outdoors, close to water	decay/mummification/skeletonization	approximately 4 months	Spychała et al. 2026 a
Ascomycota	*Yarrowia deformans*	Poland	outdoors, close to water	decay/mummification/skeletonization	approximately 4 months	Spychała et al. 2026 a
Ascomycota	*Yarrowia deformans*	Poland	outdoors, close to water	decay/mummification/skeletonization	approximately 4 months	Spychała et al. 2026 a
Ascomycota	*Yarrowia deformans*	Poland	outdoors, close to water	decay/mummification/skeletonization	approximately 4 months	Spychała et al. 2026 a
Ascomycota	*Yarrowia deformans*	Poland	outdoors, close to water	decay/mummification/skeletonization	approximately 4 months	Spychała et al. 2026 a
Ascomycota	*Yarrowia deformans*	Poland	outdoors, close to water	decay/mummification/skeletonization	approximately 4 months	Spychała et al. 2026 a
Ascomycota	*Yarrowia deformans*	Poland	outdoors, close to water	decay/mummification/skeletonization	approximately 4 months	Spychała et al. 2026 a
Ascomycota	*Yarrowia deformans* (Zach) M. Groenew. & M.T. Sm.	Poland	outdoors	advanced decay	not indicated	Spychała, K 2025
Ascomycota	*Yarrowia deformans* (Zach) M. Groenew. & M.T. Sm.	Poland	outdoors	Fresh stage	-	Spychała et al. 2026 b
Ascomycota	*Yarrowia deformans* (Zach) M. Groenew. & M.T. Sm.	Poland	outdoors	Fresh stage	-	Spychała et al. 2026 b
Ascomycota	*Yarrowia deformans* (Zach) M. Groenew. & M.T. Sm.	Poland	outdoors	Fresh stage	-	Spychała et al. 2026 b
Ascomycota	*Yarrowia deformans* (Zach) M. Groenew. & M.T. Sm.	Poland	outdoors	Advanced decay	-	Spychała et al. 2026 b
Ascomycota	*Yarrowia deformans* (Zach) M. Groenew. & M.T. Sm.	Poland	outdoors	Advanced decay	-	Spychała et al. 2026 b
Ascomycota	*Yarrowia deformans* (Zach) M. Groenew. & M.T. Sm.	Poland	outdoors	Advanced decay	-	Spychała et al. 2026 b
Ascomycota	*Yarrowia deformans* (Zach) M. Groenew. & M.T. Sm.	Poland	outdoors	Advanced decay	-	Spychała et al. 2026 b
Ascomycota	*Yarrowia deformans* (Zach) M. Groenew. & M.T. Sm.	Poland	outdoors	Advanced decay	-	Spychała et al. 2026 b
Ascomycota	*Yarrowia deformans* (Zach) M. Groenew. & M.T. Sm.	Poland	outdoors	Advanced decay	-	Spychała et al. 2026 b
Ascomycota	*Yarrowia deformans* (Zach) M. Groenew. & M.T. Sm.	Poland	outdoors	Advanced decay	-	Spychała et al. 2026 b
Ascomycota	*Yarrowia galli* (G. Péter, Dlauchy, Vasdinyei, Tornai-Leh. & T. Deák) Gouliam., R.A. Dimitrov, M.T. Sm. & M. Groenew.	Poland	outdoors	Fresh stage	-	Spychała et al. 2026 b
Ascomycota	*Yarrowia lipolytica*	Poland	outdoors, close to water	decay/mummification/skeletonization	approximately 4 months	Spychała et al. 2026 a
Ascomycota	*Yarrowia lipolytica*	Poland	outdoors, close to water	decay/mummification/skeletonization	approximately 4 months	Spychała et al. 2026 a
Ascomycota	*Yarrowia lipolytica*	Poland	outdoors, close to water	decay/mummification/skeletonization	approximately 4 months	Spychała et al. 2026 a
Ascomycota	*Yarrowia lipolytica*	Poland	outdoors, close to water	decay/mummification/skeletonization	approximately 4 months	Spychała et al. 2026 a
Ascomycota	*Yarrowia lipolytica*	Poland	outdoors, close to water	decay/mummification/skeletonization	approximately 4 months	Spychała et al. 2026 a
Ascomycota	*Yarrowia lipolytica*	Poland	outdoors, close to water	decay/mummification/skeletonization	approximately 4 months	Spychała et al. 2026 a
Ascomycota	*Yarrowia lipolytica*	Poland	outdoors, close to water	decay/mummification/skeletonization	approximately 4 months	Spychała et al. 2026 a
Ascomycota	*Yarrowia lipolytica*	Poland	outdoors, close to water	decay/mummification/skeletonization	approximately 4 months	Spychała et al. 2026 a
Ascomycota	*Yarrowia lipolytica*	Poland	outdoors, close to water	decay/mummification/skeletonization	approximately 4 months	Spychała et al. 2026 a
Ascomycota	*Yarrowia lipolytica*	Poland	outdoors, close to water	decay/mummification/skeletonization	approximately 4 months	Spychała et al. 2026 a
Ascomycota	*Yarrowia lipolytica*	Poland	outdoors, close to water	decay/mummification/skeletonization	approximately 4 months	Spychała et al. 2026 a
Ascomycota	*Yarrowia lipolytica* (Wick., Kurtzman & Herman) Van der Walt	Poland	outdoors	advanced decay	not indicated	Spychała, K 2025
Ascomycota	*Yarrowia lipolytica* (Wick., Kurtzman & Herman) Van der Walt & Arx	Germany	indoors	advanced decay	17 days	Piepenbring, M 2025
Ascomycota	*Yarrowia lipolytica* (Wick., Kurtzman & Herman) Van der Walt & Arx	Germany	indoors	advanced decay	not indicated	Schwarz et al. 2015
Ascomycota	*Yarrowia lipolytica* (Wick., Kurtzman & Herman) Van der Walt & Arx	Poland	outdoors	Fresh stage	-	Spychała et al. 2026 b
Ascomycota	*Yarrowia lipolytica* (Wick., Kurtzman & Herman) Van der Walt & Arx	Poland	outdoors	Fresh stage	-	Spychała et al. 2026 b
Ascomycota	*Yarrowia lipolytica* (Wick., Kurtzman & Herman) Van der Walt & Arx	Poland	outdoors	Fresh stage	-	Spychała et al. 2026 b
Ascomycota	*Yarrowia lipolytica* (Wick., Kurtzman & Herman) Van der Walt & Arx	Poland	outdoors	Advanced decay	-	Spychała et al. 2026 b
Ascomycota	*Yarrowia lipolytica* (Wick., Kurtzman & Herman) Van der Walt & Arx	Poland	outdoors	Advanced decay	-	Spychała et al. 2026 b
Ascomycota	*Yarrowia lipolytica* (Wick., Kurtzman & Herman) Van der Walt & Arx	Poland	outdoors	Advanced decay	-	Spychała et al. 2026 b
Ascomycota	*Yarrowia lipolytica* (Wick., Kurtzman & Herman) Van der Walt & Arx	Poland	outdoors	Advanced decay	-	Spychała et al. 2026 b
Ascomycota	*Yarrowia lipolytica* (Wick., Kurtzman & Herman) Van der Walt & Arx	Poland	outdoors	Advanced decay	-	Spychała et al. 2026 b
Ascomycota	*Yarrowia lipolytica* (Wick., Kurtzman & Herman) Van der Walt & Arx	Poland	outdoors	Advanced decay	-	Spychała et al. 2026 b
Ascomycota	*Yarrowia lipolytica* (Wick., Kurtzman & Herman) Van der Walt & Arx	Poland	outdoors	Fresh stage	-	Spychała et al. 2026 b
Ascomycota	*Yarrowia lipolytica* (Wick., Kurtzman & Herman) Van der Walt & Arx	Poland	outdoors	Fresh stage	-	Spychała et al. 2026 b
Ascomycota	*Yarrowia lipolytica* (Wick., Kurtzman & Herman) Van der Walt & Arx	Poland	outdoors	Fresh stage	-	Spychała et al. 2026 b
Ascomycota	*Yarrowia lipolytica* (Wick., Kurtzman & Herman) Van der Walt & Arx	Poland	outdoors	Fresh stage	-	Spychała et al. 2026 b
Ascomycota	*Yarrowia lipolytica* (Wick., Kurtzman & Herman) Van der Walt & Arx	Poland	outdoors	Fresh stage	-	Spychała et al. 2026 b
Ascomycota	*Yarrowia lipolytica* (Wick., Kurtzman & Herman) Van der Walt & Arx	Poland	outdoors	Advanced decay	-	Spychała et al. 2026 b
Ascomycota	*Yarrowia lipolytica* (Wick., Kurtzman & Herman) Van der Walt & Arx	Poland	outdoors	Advanced decay	-	Spychała et al. 2026 b
Ascomycota	*Yarrowia lipolytica* (Wick., Kurtzman & Herman) Van der Walt & Arx	Poland	outdoors	Advanced decay	-	Spychała et al. 2026 b
Ascomycota	*Yarrowia lipolytica* (Wick., Kurtzman & Herman) Van der Walt & Arx	Poland	outdoors	Advanced decay	-	Spychała et al. 2026 b
Ascomycota	*Yarrowia lipolytica* (Wick., Kurtzman & Herman) Van der Walt & Arx	Poland	outdoors	Advanced decay	-	Spychała et al. 2026 b
Ascomycota	*Yarrowia lipolytica* (Wick., Kurtzman & Herman) Van der Walt & Arx	Poland	outdoors	Advanced decay	-	Spychała et al. 2026 b
Ascomycota	*Yarrowia lipolytica* (Wick., Kurtzman & Herman) Van der Walt & Arx	Poland	outdoors	Advanced decay	-	Spychała et al. 2026 b
Ascomycota	*Yarrowia lipolytica* (Wick., Kurtzman & Herman) Van der Walt & Arx	Poland	outdoors	Advanced decay	-	Spychała et al. 2026 b
Ascomycota	*Yarrowia lipolytica* (Wick., Kurtzman & Herman) Van der Walt & Arx	Poland	outdoors	Fresh stage	-	Spychała et al. 2026 b
Ascomycota	*Yarrowia lipolytica* (Wick., Kurtzman & Herman) Van der Walt & Arx	Poland	outdoors	Advanced postmortem changes	-	Spychała et al. 2026 b
Ascomycota	*Yarrowia lipolytica* (Wick., Kurtzman & Herman) Van der Walt & Arx	Poland	outdoors	Advanced postmortem changes	-	Spychała et al. 2026 b
Ascomycota	*Yarrowia lipolytica* (Wick., Kurtzman & Herman) Van der Walt & Arx	Poland	outdoors	Advanced postmortem changes	-	Spychała et al. 2026 b
Ascomycota	*Yarrowia lipolytica* (Wick., Kurtzman & Herman) Van der Walt & Arx	Poland	outdoors	Advanced postmortem changes	-	Spychała et al. 2026 b
Ascomycota	*Yarrowia lipolytica* (Wick., Kurtzman & Herman) Van der Walt & Arx	Poland	outdoors	Advanced postmortem changes	-	Spychała et al. 2026 b
Ascomycota	*Yarrowia lipolytica* (Wick., Kurtzman & Herman) Van der Walt & Arx	Poland	outdoors	Advanced postmortem changes	-	Spychała et al. 2026 b
Ascomycota	*Yarrowia lipolytica* (Wick., Kurtzman & Herman) Van der Walt & Arx	Poland	outdoors	Decay with signs of mummification	-	Spychała et al. 2026 b
Ascomycota	*Yarrowia lipolytica* (Wick., Kurtzman & Herman) Van der Walt & Arx	Poland	outdoors	Decay with signs of mummification	-	Spychała et al. 2026 b
Ascomycota	*Yarrowia lipolytica* (Wick., Kurtzman & Herman) Van der Walt & Arx	Poland	outdoors	Decay with signs of mummification	-	Spychała et al. 2026 b
Ascomycota	*Yarrowia lipolytica* (Wick., Kurtzman & Herman) Van der Walt & Arx	Poland	outdoors	Decay with signs of mummification	-	Spychała et al. 2026 b
Ascomycota	*Yarrowia lipolytica* (Wick., Kurtzman & Herman) Van der Walt & Arx	Poland	outdoors	Decay with signs of mummification	-	Spychała et al. 2026 b
Ascomycota	*Yarrowia lipolytica* (Wick., Kurtzman & Herman) Van der Walt & Arx	Poland	outdoors	Decay with signs of mummification	-	Spychała et al. 2026 b
Ascomycota	*Yarrowia lipolytica* (Wick., Kurtzman & Herman) Van der Walt & Arx	Poland	outdoors	Decay with signs of mummification	-	Spychała et al. 2026 b
Ascomycota	*Yarrowia lipolytica* (Wick., Kurtzman & Herman) Van der Walt & Arx	Poland	outdoors	Decay with signs of mummification	-	Spychała et al. 2026 b
Ascomycota	*Yarrowia lipolytica* (Wick., Kurtzman & Herman) Van der Walt & Arx	Poland	outdoors	Decay with signs of mummification	-	Spychała et al. 2026 b
Ascomycota	*Yarrowia lipolytica* (Wick., Kurtzman & Herman) Van der Walt & Arx	Poland	outdoors	Decay with signs of mummification	-	Spychała et al. 2026 b
Ascomycota	*Yarrowia lipolytica* (Wick., Kurtzman & Herman) Van der Walt & Arx	Poland	outdoors	Decay with signs of mummification	-	Spychała et al. 2026 b
Ascomycota	*Yarrowia lipolytica* (Wick., Kurtzman & Herman) Van der Walt & Arx	Poland	outdoors	Decay with signs of mummification	-	Spychała et al. 2026 b
Ascomycota	*Yarrowia lipolytica* (Wick., Kurtzman & Herman) Van der Walt & Arx	Poland	outdoors	Decay with signs of mummification	-	Spychała et al. 2026 b
Ascomycota	*Yarrowia lipolytica* (Wick., Kurtzman & Herman) Van der Walt & Arx	Poland	outdoors	Decay with signs of mummification	-	Spychała et al. 2026 b
Ascomycota	*Yarrowia lipolytica* (Wick., Kurtzman & Herman) Van der Walt & Arx	Poland	outdoors	Decay with signs of mummification	-	Spychała et al. 2026 b
Ascomycota	*Yarrowia parophoni* Gouliam., R.A. Dimitrov, Guéorguiev, M.T. Sm. & M. Groenew.	Poland	outdoors	Advanced decay	-	Spychała et al. 2026 b
Ascomycota	*Yarrowia parophoni* Gouliam., R.A. Dimitrov, Guéorguiev, M.T. Sm. & M. Groenew.	Poland	outdoors	Advanced decay	-	Spychała et al. 2026 b
Ascomycota	*Yarrowia parophoni* Gouliam., R.A. Dimitrov, Guéorguiev, M.T. Sm. & M. Groenew.	Poland	outdoors	Advanced decay	-	Spychała et al. 2026 b
Ascomycota	*Yarrowia parophoni* Gouliam., R.A. Dimitrov, Guéorguiev, M.T. Sm. & M. Groenew.	Poland	outdoors	Advanced decay	-	Spychała et al. 2026 b
Ascomycota	*Yarrowia parophoni* Gouliam., R.A. Dimitrov, Guéorguiev, M.T. Sm. & M. Groenew.	Poland	outdoors	Advanced decay	-	Spychała et al. 2026 b
Ascomycota	*Yarrowia parophoni* Gouliam., R.A. Dimitrov, Guéorguiev, M.T. Sm. & M. Groenew.	Poland	outdoors	Fresh stage	-	Spychała et al. 2026 b
Basidiomycota	*Apiotrichum dulcitum* (Berkhout) Yurkov & Boekhout	Poland	outdoors	Advanced decay	-	Spychała et al. 2026 b
Basidiomycota	*Apiotrichum dulcitum* (Berkhout) Yurkov & Boekhout	Poland	outdoors	Advanced decay	-	Spychała et al. 2026 b
Basidiomycota	*Botryobasidium capitatum* (Link) Rossman & W.C. Allen	Italy	outdoors, buried	mummification/skeletonization	not indicated	Ciferri R 1943
Basidiomycota	*Bullera alba* (W.F. Hanna) Derx	Poland	outdoors	Fresh stage	-	Spychała et al. 2026 b
Basidiomycota	*Bullera alba* (W.F. Hanna) Derx	Poland	outdoors	Fresh stage	-	Spychała et al. 2026 b
Basidiomycota	*Bullera alba* (W.F. Hanna) Derx	Poland	outdoors	Fresh stage	-	Spychała et al. 2026 b
Basidiomycota	*Cryptococcus* sp.	Iraq	indoors	not indicated	not indicated (long)	Hammad et al. 2002
Basidiomycota	*Ctenomyces vellereus* (Sacc. & Speg.) P.M. Kirk	Italy	outdoors, buried	mummification/skeletonization	not indicated	Bianchini e Manfrini 1924
Basidiomycota	*Haplotrichum* sp.	Germany	outdoors, buried	not indicated	3 days	Dósa 1955
Basidiomycota	*Haplotrichum* sp.	Germany	outdoors, buried	not indicated	3 months	Dósa 1955
Basidiomycota	*Haplotrichum* sp.	Germany	outdoors, buried	not indicated	3 months	Dósa 1955
Basidiomycota	*Hypholoma fasciculare* (Huds.) P. Kumm.	Germany	indoors	advanced decay	> 30 days	Piepenbring, M 2025
Basidiomycota	*Hypholoma fasciculare* (Huds.) P. Kumm.	Germany	indoors	advanced decay	> 30 days	Piepenbring, M 2025
Basidiomycota	*Rhodotorula glutinis* (Fresen.) F.C. Harrison, Trans. Roy. Soc. Canada, Sect. 5	Poland	outdoors	Fresh stage	-	Spychała et al. 2026 b
Basidiomycota	*Rhodotorula mucilaginosa* (A. Jörg.) F.C. Harrison	Germany	outdoors, buried	not indicated	2 months	Dósa 1955
Basidiomycota	*Rhodotorula mucilaginosa* (A. Jörg.) F.C. Harrison	Germany	outdoors, buried	not indicated	2 years	Dósa 1955
Basidiomycota	*Rhodotorula mucilaginosa* (A. Jörg.) F.C. Harrison	Germany	outdoors, buried	not indicated	3 months	Dósa 1955
Basidiomycota	*Rhodotorula mucilaginosa* (A. Jörg.) F.C. Harrison	Germany	outdoors, buried	not indicated	2 weeks	Dósa 1955
Basidiomycota	*Rhodotorula mucilaginosa* (A. Jörg.) F.C. Harrison	Germany	outdoors, buried	not indicated	3 months	Dósa 1955
Basidiomycota	*Rhodotorula mucilaginosa* (A. Jörg.) F.C. Harrison	Poland	outdoors	Fresh stage	-	Spychała et al. 2026 b
Basidiomycota	*Rhodotorula mucilaginosa* (A. Jörg.) F.C. Harrison	Poland	outdoors	Fresh stage	-	Spychała et al. 2026 b
Basidiomycota	*Rhodotorula mucilaginosa* (A. Jörg.) F.C. Harrison	Poland	outdoors	Fresh stage	-	Spychała et al. 2026 b
Basidiomycota	*Rhodotorula mucilaginosa* (A. Jörg.) F.C. Harrison	Poland	outdoors	Fresh stage	-	Spychała et al. 2026 b
Basidiomycota	*Rhodotorula mucilaginosa* (A. Jörg.) F.C. Harrison	Poland	outdoors	Fresh stage	-	Spychała et al. 2026 b
Basidiomycota	*Rhodotorula mucilaginosa* (A. Jörg.) F.C. Harrison	Poland	outdoors	Fresh stage	-	Spychała et al. 2026 b
Basidiomycota	*Rhodotorula mucilaginosa* (A. Jörg.) F.C. Harrison	Poland	outdoors	Fresh stage	-	Spychała et al. 2026 b
Basidiomycota	*Trichosporon* sp.	Brazil	indoors	fresh	not indicated	Sidrim et al. 2010
Basidiomycota	*Trichosporon* sp.	Brazil	outdoors	mummification/skeletonization	not indicated	Sidrim et al. 2010
Basidiomycota	*Trichosporon* sp.	Brazil	indoors	fresh	not indicated	Sidrim et al. 2010
Dictyosteliomycota	*Dictyostelium mucoroides* Bref.	Italy	outdoors, buried	mummification/skeletonization	not indicated	Bianchini e Manfrini 1924
Mortierellomycota	*Linnemannia hyalina*	Poland	outdoors, close to water	decay/mummification/skeletonization	approximately 4 months	Spychała et al. 2026 a
Mortierellomycota	*Linnemannia hyalina* (Harz) Vandepol & Bonito	Poland	outdoors	Advanced decay	-	Spychała et al. 2026 b
Mortierellomycota	*Linnemannia hyalina* (Harz) Vandepol & Bonito	Poland	outdoors	Advanced decay	-	Spychała et al. 2026 b
Mortierellomycota	*Linnemannia hyalina* (Harz) Vandepol & Bonito	Poland	outdoors	Advanced decay	-	Spychała et al. 2026 b
Mucoromycota	*Alternaria infectoria* E.G. Simmons	Poland	outdoors	Advanced postmortem changes	-	Spychała et al. 2026 b
Mucoromycota	*Chlamydomucor racemosus* Bref.	Germany	outdoors, buried	not indicated	2 months	Dósa 1955
Mucoromycota	*Chlamydomucor racemosus* Bref.	Germany	outdoors, buried	not indicated	3 months	Dósa 1955
Mucoromycota	*Chlamydomucor racemosus* Bref.	Germany	outdoors, buried	not indicated	3 months	Dósa 1955
Mucoromycota	*Chlamydomucor racemosus* Bref.	Germany	outdoors, buried	not indicated	1 month	Dósa 1955
Mucoromycota	*Chlamydomucor racemosus* Bref.	Germany	indoors	advanced decay	not indicated	Schwarz et al. 2015
Mucoromycota	*Chlamydomucor racemosus* Bref.	Italy	outdoors, buried	fresh/decay	not indicated	Ciferri R 1943
Mucoromycota	*Chlamydomucor racemosus* Bref.	Italy	-	fresh/decay	not indicated	Bianchini, G 1923
Mucoromycota	*Chlamydomucor racemosus* Bref.	Poland	outdoors	advanced decay	not indicated	Spychała, K 2025
Mucoromycota	*Chlamydomucor racemosus* Bref.	Poland	outdoors	Fresh stage	-	Spychała et al. 2026 b
Mucoromycota	*Helicostylum elegans* Corda	Germany	indoors	decay/mummification/skeletonization	not indicated	Schwarz et al. 2015
Mucoromycota	*Helicostylum pulchrum* (Preuss) Pidopl. & Milko	Poland	outdoors	advanced decay	not indicated	Spychała, K 2025
Mucoromycota	*Lichtheimia corymbifera* (Cohn) Vuill.	Germany	outdoors, buried	not indicated	3 days	Dósa 1955
Mucoromycota	*Lichtheimia corymbifera* (Cohn) Vuill.	Germany	outdoors, buried	not indicated	2 months	Dósa 1955
Mucoromycota	*Lichtheimia corymbifera* (Cohn) Vuill.	Germany	outdoors, buried	not indicated	3 months	Dósa 1955
Mucoromycota	*Lichtheimia corymbifera* (Cohn) Vuill.	Germany	outdoors, buried	not indicated	2 weeks	Dósa 1955
Mucoromycota	*Lichtheimia corymbifera* (Cohn) Vuill.	Germany	outdoors, buried	not indicated	3 months	Dósa 1955
Mucoromycota	*Lichtheimia corymbifera* (Cohn) Vuill.	Germany	indoors	advanced decay	not indicated	Schwarz et al. 2015
Mucoromycota	*Mortierella polycephala* Coem.	Poland	outdoors	advanced decay	not indicated	Spychała, K 2025
Mucoromycota	*Mortierella* sp.	Holland	indoors	not indicated	several weeks	van de Voorde & van Dijck 1982
Mucoromycota	*Mucor circinelloides*	Poland	outdoors, close to water	decay/mummification/skeletonization	approximately 4 months	Spychała et al. 2026 a
Mucoromycota	*Mucor circinelloides*	Poland	outdoors, close to water	decay/mummification/skeletonization	approximately 4 months	Spychała et al. 2026 a
Mucoromycota	*Mucor circinelloides* Tiegh.	Germany	indoors	fresh/decay	>12 days	Piepenbring, M 2025
Mucoromycota	*Mucor circinelloides* Tiegh.	Germany	outdoors, buried	decay/mummification/skeletonization	5 weeks	Schwarz et al. 2015
Mucoromycota	*Mucor circinelloides* Tiegh.	Germany	outdoors, buried	decay/mummification/skeletonization	10 weeks	Schwarz et al. 2015
Mucoromycota	*Mucor circinelloides* Tiegh., Ann. Sci. Nat., Bot. sér. 6	Poland	outdoors	Advanced decay	-	Spychała et al. 2026 b
Mucoromycota	*Mucor circinelloides* Tiegh., Ann. Sci. Nat., Bot. sér. 6	Poland	outdoors	Advanced decay	-	Spychała et al. 2026 b
Mucoromycota	*Mucor circinelloides* Tiegh., Ann. Sci. Nat., Bot. sér. 6	Poland	outdoors	Advanced decay	-	Spychała et al. 2026 b
Mucoromycota	*Mucor circinelloides* Tiegh., Ann. Sci. Nat., Bot. sér. 6	Poland	outdoors	Advanced decay	-	Spychała et al. 2026 b
Mucoromycota	*Mucor flavus*	Poland	outdoors, close to water	decay/mummification/skeletonization	approximately 4 months	Spychała et al. 2026 a
Mucoromycota	*Mucor flavus*	Poland	outdoors, close to water	decay/mummification/skeletonization	approximately 4 months	Spychała et al. 2026 a
Mucoromycota	*Mucor heterogamus* Vuill.	Germany	outdoors, buried	advanced decay	121 days	Piepenbring, M 2025
Mucoromycota	*Mucor heterosporus* Fisch	Italy	outdoors, buried	mummification/skeletonization	not indicated	Bianchini e Manfrini 1924
Mucoromycota	*Mucor hiemalis*	Poland	outdoors, close to water	decay/mummification/skeletonization	approximately 4 months	Spychała et al. 2026 a
Mucoromycota	*Mucor hiemalis*	Poland	outdoors, close to water	decay/mummification/skeletonization	approximately 4 months	Spychała et al. 2026 a
Mucoromycota	*Mucor hiemalis*	Poland	outdoors, close to water	decay/mummification/skeletonization	approximately 4 months	Spychała et al. 2026 a
Mucoromycota	*Mucor hiemalis*	Poland	outdoors, close to water	decay/mummification/skeletonization	approximately 4 months	Spychała et al. 2026 a
Mucoromycota	*Mucor hiemalis*	Poland	outdoors, close to water	decay/mummification/skeletonization	approximately 4 months	Spychała et al. 2026 a
Mucoromycota	*Mucor hiemalis*	Poland	outdoors, close to water	decay/mummification/skeletonization	approximately 4 months	Spychała et al. 2026 a
Mucoromycota	*Mucor hiemalis*	Poland	outdoors, close to water	decay/mummification/skeletonization	approximately 4 months	Spychała et al. 2026 a
Mucoromycota	*Mucor hiemalis* Wehmer	Germany	indoors	fresh	13 days	Piepenbring, M 2025
Mucoromycota	*Mucor hiemalis* Wehmer	Germany	outdoors	advanced decay	>11 days	Piepenbring, M 2025
Mucoromycota	*Mucor hiemalis* Wehmer	Germany	outdoors	fresh	7 days	Piepenbring, M 2025
Mucoromycota	*Mucor hiemalis* Wehmer	Italy	outdoors	decay/mummification/skeletonization	6 days	Bellini et al. 2015
Mucoromycota	*Mucor hiemalis* Wehmer	Italy	outdoors	decay/mummification/skeletonization	6 days	Bellini et al. 2015
Mucoromycota	*Mucor hiemalis* Wehmer	Poland	outdoors	Fresh stage	-	Spychała et al. 2026 b
Mucoromycota	*Mucor hiemalis* Wehmer	Poland	outdoors	Fresh stage	-	Spychała et al. 2026 b
Mucoromycota	*Mucor hiemalis* Wehmer	Poland	outdoors	Fresh stage	-	Spychała et al. 2026 b
Mucoromycota	*Mucor hiemalis* Wehmer	Poland	outdoors	Fresh stage	-	Spychała et al. 2026 b
Mucoromycota	*Mucor hiemalis* Wehmer	Poland	outdoors	Advanced decay	-	Spychała et al. 2026 b
Mucoromycota	*Mucor hiemalis* Wehmer	Poland	outdoors	Advanced decay	-	Spychała et al. 2026 b
Mucoromycota	*Mucor hiemalis* Wehmer	Poland	outdoors	Decay with signs of mummification	-	Spychała et al. 2026 b
Mucoromycota	*Mucor hiemalis* Wehmer	Poland	outdoors	Decay with signs of mummification	-	Spychała et al. 2026 b
Mucoromycota	*Mucor hiemalis* Wehmer	Poland	outdoors	Decay with signs of mummification	-	Spychała et al. 2026 b
Mucoromycota	*Mucor hiemalis* Wehmer	Poland	outdoors	Decay with signs of mummification	-	Spychała et al. 2026 b
Mucoromycota	*Mucor hiemalis* Wehmer	UK	not indicated	not indicated	not indicated	Hawksworth & Wiltshire 2015
Mucoromycota	*Mucor mucedo*	Poland	outdoors, close to water	decay/mummification/skeletonization	approximately 4 months	Spychała et al. 2026 a
Mucoromycota	*Mucor plumbeus* Bonord.	Germany	indoors	fresh	17 days	Piepenbring, M 2025
Mucoromycota	*Mucor plumbeus* Bonord.	Germany	indoors	advanced decay	8 days	Piepenbring, M 2025
Mucoromycota	*Mucor plumbeus* Bonord.	Germany	outdoors, water	decay/mummification/skeletonization	not indicated	Schwarz et al. 2015
Mucoromycota	*Mucor plumbeus* Bonord.	Germany	indoors	advanced decay	not indicated	Schwarz et al. 2015
Mucoromycota	*Mucor plumbeus* Bonord.	Germany	-	advanced decay	not indicated	Schwarz et al. 2015
Mucoromycota	*Mucor plumbeus* Bonord.	Germany	indoors	decay/mummification/skeletonization	not indicated	Schwarz et al. 2015
Mucoromycota	*Mucor plumbeus* Bonord.	Poland	outdoors	Advanced postmortem changes	-	Spychała et al. 2026 b
Mucoromycota	*Mucor plumbeus* Bonord.	Poland	outdoors	Advanced postmortem changes	-	Spychała et al. 2026 b
Mucoromycota	*Mucor plumbeus* Bonord.	Poland	outdoors	Advanced postmortem changes	-	Spychała et al. 2026 b
Mucoromycota	*Mucor plumbeus* Bonord.	Poland	outdoors	Advanced postmortem changes	-	Spychała et al. 2026 b
Mucoromycota	*Mucor plumbeus* Bonord.	UK	not indicated	not indicated	not indicated	Hawksworth & Wiltshire 2015
Mucoromycota	*Mucor pseudolusitanicus* L. Wagner & G. Walther	Germany	outdoors	advanced decay	>10 days	Piepenbring, M 2025
Mucoromycota	*Mucor saturninus* Hagem	Germany	indoors	advanced decay	not indicated	Schwarz et al. 2015
Mucoromycota	*Mucor saturninus* Hagem	Germany	indoors	advanced decay	not indicated	Schwarz et al. 2015
Mucoromycota	*Mucor saturninus* Hagem	Poland	outdoors	Advanced postmortem changes	-	Spychała et al. 2026 b
Mucoromycota	*Mucor saturninus* Hagem.	Italy	indoors	advanced decay	-	Iannaccone et al. 2026 current
Mucoromycota	*Mucor* sp.	Brazil	indoors	fresh	not indicated	Sidrim et al. 2010
Mucoromycota	*Mucor* sp.	Brazil	indoors	fresh	not indicated	Sidrim et al. 2010
Mucoromycota	*Mucor* sp.	Brazil	indoors	fresh	not indicated	Sidrim et al. 2010
Mucoromycota	*Mucor* sp.	Brazil	outdoors	mummification/skeletonization	not indicated	Sidrim et al. 2010
Mucoromycota	*Mucor* sp.	Germany	outdoors, buried	not indicated	2 months	Dósa 1955
Mucoromycota	*Mucor* sp.	Germany	outdoors, buried	not indicated	48 days	Dósa 1955
Mucoromycota	*Mucor* sp.	Germany	outdoors, buried	not indicated	10 days	Dósa 1955
Mucoromycota	*Mucor* sp.	Germany	outdoors, buried	not indicated	1 month	Dósa 1955
Mucoromycota	*Mucor* sp.	Japan	outdoors	advanced decay	1 month	Kato et al. 2023
Mucoromycota	*Mucor* sp.	UK	outdoors, water	not indicated	at least 5 weeks in water	Hawksworth & Wiltshire 2011
Mucoromycota	*Thelactis flava* H. Mart.	Germany	indoors	decay/mummification/skeletonization	not indicated	Schwarz et al. 2015
Mucoromycota	*Thelactis flava* H. Mart.	Germany	indoors	advanced decay	2 months	Schwarz et al. 2015
Oomycota	*Pythium* sp.	Switzerland	outdoors, buried	mummification/skeletonization	more than 200 years	Piepenbrink 1986
Oomycota	*Pythium* sp.	UK	outdoors, water	not indicated	at least 5 weeks in water	Hawksworth & Wiltshire 2011

## Data Availability

The nucleotide sequences generated in this study have been deposited in GenBank under the following accession numbers: PZ675949, PZ675950, PZ675951, PZ675955, PZ675956, PZ675957, PZ707411, and PZ707412 (see Table 1 for the correspondence between isolates, markers, and accession numbers). The updated checklist of cadaver-associated culturable fungi is provided in Table 3.
